# Gene Expression Patterns during Light and Dark Infection of *Prochlorococcus* by Cyanophage

**DOI:** 10.1371/journal.pone.0165375

**Published:** 2016-10-27

**Authors:** Luke R. Thompson, Qinglu Zeng, Sallie W. Chisholm

**Affiliations:** 1 Department of Biology, Massachusetts Institute of Technology, Cambridge, Massachusetts, United States of America; 2 Department of Civil and Environmental Engineering, Massachusetts Institute of Technology, Cambridge, Massachusetts, United States of America; Mount Allison University, CANADA

## Abstract

Cyanophage infecting the marine cyanobacteria *Prochlorococcus* and *Synechococcus* require light and host photosystem activity for optimal reproduction. Many cyanophages encode multiple photosynthetic electron transport (PET) proteins, which are presumed to maintain electron flow and produce ATP and NADPH for nucleotide biosynthesis and phage genome replication. However, evidence suggests phage augment NADPH production via the pentose phosphate pathway (PPP), thus calling into question the need for NADPH production by PET. Genes implicated in cyclic PET have since been identified in cyanophage genomes. It remains an open question which mode of PET, cyclic or linear, predominates in infected cyanobacteria, and thus whether the balance is towards producing ATP or NADPH. We sequenced transcriptomes of a cyanophage (P-HM2) and its host (*Prochlorococcus* MED4) throughout infection in the light or in the dark, and analyzed these data in the context of phage replication and metabolite measurements. Infection was robust in the light, but phage were not produced in the dark. Host gene transcripts encoding high-light inducible proteins and two terminal oxidases (plastoquinol terminal oxidase and cytochrome *c* oxidase)—implicated in protecting the photosynthetic membrane from light stress—were the most enriched in light but not dark infection. Among the most diminished transcripts in both light and dark infection was ferredoxin–NADP^+^ reductase (FNR), which uses the electron acceptor NADP^+^ to generate NADPH in linear photosynthesis. The phage gene for CP12, which putatively inhibits the Calvin cycle enzyme that receives NADPH from FNR, was highly expressed in light infection. Therefore, both PET production of NADPH and its consumption by carbon fixation are putatively repressed during phage infection in light. Transcriptomic evidence is thus consistent with cyclic photophosphorylation using oxygen as the terminal electron acceptor as the dominant mode of PET under infection, with ATP from PET and NADPH from the PPP producing the energy and reducing equivalents for phage nucleotide biosynthesis and replication.

## Introduction

Cyanophage are viruses that infect cyanobacteria [1], including the numerically dominant marine picocyanobacteria *Prochlorococcus* and *Synechococcus* [2]. Cyanophage genomes contain various ‘auxiliary metabolic genes’ (AMGs), which are not universal but evolutionarily conserved and are thought to boost and redirect host metabolism during infection [3, 4]. Although AMGs are more abundant in the larger T4-like cyanomyophage genomes (the subject of this study) [5], they are also found in T7-like cyanopodophages [6, 7], and host homologs of AMGs are induced by cyanosiphophages [8], suggesting a common infection strategy across all three cyanophage families.

As evidenced by the functions of proteins encoded by AMGs—and by the high demand for nucleotides for phage replication—nucleotide biosynthesis is a key product of cyanophage-infected host metabolism; indeed, nucleotide biosynthesis genes are common in cyanophage genomes [6, 9]. The amount of nucleotides required for a typical cyanophage ‘burst’ exceeds the size of the host chromosome, and therefore we postulated that cyanophage synthesize most of their nucleotides *de novo* [4]. The synthesis of deoxynucleoside triphosphates (shortened to ‘nucleotides’ here) requires phosphate—potentially accounting for the presence of phosphate-acquisition genes in some cyanophages [6, 10, 11]—but is also energy-intensive, requiring large amounts of ATP (energy) and NADPH (reducing equivalents).

Photosynthesis is the primary source of ATP and NADPH for cyanobacteria and—based on experimental evidence and the AMGs carried by cyanophage—also for cyanophage. Light increases phage burst size in cyanophage infections [12], and both light and photosynthesis are required for maximal phage production [13–15]; light is even required for adsorption of some cyanophages [16, 17]. Genes encoding photosynthetic light capture and electron transport are widespread in cyanophage genomes [6, 18–20], and phage photosystem II and high-light inducible proteins are detected in the host during infection [15]. Host genes encoding high-light inducible proteins are induced during infection in the light [21, 22], further suggesting maintenance of photosynthetic electron flow [23], possibly to counteract photo-oxidative damage induced by cyanophage [24]. However, while both host and phage express genes for the ‘light reactions’ of photosynthesis, genes for the Calvin cycle are not found in cyanophage genomes. In fact they often carry genes orthologous to the host gene *cp12*, the product of which inhibits the Calvin Cycle [25]. In addition, cyanophages often carry AMGs that encode enzymes involved in the pentose phosphate pathway [6], which intersects with the Calvin cycle but runs in the opposite direction [4].

The above observations led to the hypothesis that the light reactions of photosynthesis and the pentose phosphate pathway are activated during infection, but the Calvin Cycle is inhibited. This would enable ATP and NADPH produced by the first two processes to be used for nucleotide biosynthesis rather than drained by the Calvin cycle. In support of this hypothesis [4], we used qPCR to show that phage genes for the light reactions (*psbA*) and pentose phosphate pathway (*talC*, *gnd*, *zwf*) and inhibition of the Calvin cycle (*cp12*) were highly expressed during infection in the light and poorly expressed during infection in the dark. Simultaneously, the intracellular NADPH/NADP^+^ ratio doubled in infected relative to uninfected cells in the light, and increased only slightly in the dark (results from [4] are replotted in S1F Fig). Recent work using radiolabeled inorganic carbon has shown that the Calvin cycle is indeed inhibited during cyanophage infection [26].

The observed increase in NADPH/NADP^+^ in light infection, even as cyanophage were putatively consuming NADPH to synthesize nucleotides, implied that infection boosted NADPH production. However, it was difficult to quantify how much NADPH production was from phage-augmented pentose phosphate pathway activity (and Calvin cycle inhibition) because linear photosynthetic electron transport (PET), driven by light, produces ATP *and* NADPH. A clue was provided by the observation that cyanophage genomes assembled from seawater contain genes for photosystem I [20] and NAD(P)H dehydrogenase [27, 28], raising the prospect that cyclic photophosphorylation is important for cyanophage infection [29, 30].

Multiple questions remain about the effects of phage infection on light-driven electron flow in picocyanobacteria. While identifying the mechanistic changes in PET will require targeted perturbations (e.g., mutagenesis, inhibitor studies, or radiolabeled substrates as in [26]), comparative transcriptomics of cultures undergoing infection can help discriminate between competing models. Specifically, we asked the following: Are patterns of host transcription during infection more consistent with linear or cyclic electron flow, and with which terminal electron acceptors? Do the transcripts enriched or depleted indicate the possible sources of ATP and NADPH for cyanophage nucleotide biosynthesis? Are phage-dependent changes in transcription of host genes in photosynthetic electron flow dependent on light?

We analyzed phage and host transcriptomes of T4-like cyanophage P-HM2 infecting cultures of *Prochlorococcus* MED4, maintained in the light or placed in the dark at the time of infection. Samples were derived from our previous work [4], in which we examined the relative expression of only a few selected genes. Here we extend that study and show differential expression of key host PET genes during infection in light and dark. The results suggest that changes in host gene expression, induced under phage infection, lead to changes in photosynthetic electron flow toward a cellular metabolic state appearing more optimal for phage replication.

## Materials and Methods

### Growth and infection in light and dark

The infection of *Prochlorococcus* MED4 by cyanophage P-HM2 for this experiment was described in detail in [4]. Light and dark experiments were conducted on separate days; all conditions were identical except that in the dark experiment, from splitting the culture onward, bottles were maintained in complete darkness except for inoculation with phage or spent medium and sampling, which were done in dim light. In both experiments, a mid-log-phase mother culture of *Prochlorococcus* MED4 grown in constant light (90 *μ*E m^−2^s^–1^) was split into four bottles of 2 L each (final volume). Experiments were performed in duplicate: two bottles were inoculated with cyanophage P-HM2 (MOI = 1, therefore not all cells were infected), and two bottles were given an equal volume of spent medium (late-log phase, no phage). Both spent medium and phage lysate were filtered through 0.2-*μ*m polycarbonate filters (Millipore) prior to addition. Following inoculation, cultures were placed in a dark incubator or returned to the light incubator. Temperature was maintained at 19–22°C. Samples were taken at regular intervals (0, 1, 2, 4, and 8 h) for measurement of infection parameters [4]. For RNA analysis, 200 mL culture was harvested by centrifugation at 15,000x*g* for 10 min at 4°C, decanted, resuspended in 1 mL supernatant, aliquoted into microcentrifuge tubes, centrifuged again at 15,000x*g* for 5 min at 4°C, decanted, and flash frozen in liquid nitrogen and stored at –80°C. The elapsed time from the start of sampling to freezing was 30 min; therefore, the reported time points are 0.5, 1.5, 2.5, 4.5, and 8.5 h after inoculation with phage (hereafter referred to as time “post-inoculation”).

### Strand-specific RNA-Seq library preparation for Illumina sequencing

Total RNA was extracted using the Ambion mirVana RNA isolation kit; residual genomic DNA was removed using the Turbo DNA-free kit (Ambion). RNA was then concentrated with the RNA Clean & Concentrator-5 kit (Zymo Research). Total RNA (150 ng) was fragmented to the range of 60–200 nt by magnesium-catalyzed hydrolysis (40 mM Tris-Acetate, pH 8.1, 100 mM potassium acetate, 30 mM magnesium acetate) for 4 min at 83°C, and purified with the RNA Clean & Concentrator-5 kit. Strand-specific RNA-Seq libraries were prepared using a dUTP second-strand marking protocol as described previously [31]. Briefly, first-strand cDNA was synthesized from fragmented RNA with random primers (Invitrogen) and Superscript III reverse transcriptase (Invitrogen). Second-strand cDNA was synthesized using dUTP instead of dTTP. Illumina paired-end libraries were prepared from purified double-stranded cDNA (Agencourt AMPure XP beads) following recommendations by Illumina. Second strands of cDNA containing dUTP were removed with the Uracil Cleavage System (Enzymatics), and libraries were amplified with the sequencing primers. A duplex-specific nuclease (DSN)-based method [32], which has been successfully used in *Prochlorococcus* [33], was used to remove the 16S and 23S rRNAs. Illumina sequencing primers with barcodes were used to amplify the DSN treated libraries. Barcoded libraries (40) were pooled in equal proportion in one lane and paired-end sequencing was done on an Illumina HiSeq 2000 (40 nt for insert + 6 nt for barcode).

### Mapping and counting RNA-Seq reads

Reads from each sample were separated based on their barcodes and aligned to *Prochlorococcus* MED4 and cyanophage P-HM2 genomes using the Burrows–Wheeler Aligner (BWA). GenBank annotations (BX548174.1 for MED4 and NC_015284.1 for P-HM2) were used to generate SAM alignment files using BWA. SAMtools [34] and pysam were then used to calculate the number of reads perfectly aligning to the sense and antisense strands of ORFs, rRNAs, tRNAs, and intergenic regions. Paired reads were mapped separately; paired reads mapping to the same ORF were counted as one transcript for that ORF, whereas paired reads mapping to separate adjacent ORFs were counted once for each ORF. An average of 2.8 million (range 1.7–4.1 million) mapped reads were recovered from each sample. As a result of DSN treatment, the percentage of host rRNA (16S and 23S) reads in the samples was reduced to an average of 14% (range 4–59%, compared to 97% in untreated samples). Host 16S and 23S rRNA read counts were removed from count tables prior to any filtering or normalization.

### Normalization of phage and host transcript abundance

Counts were normalized per sample using the RPKM method (reads per kbp gene length per million reads). Phage transcript counts were normalized to the total of phage plus host transcript counts (per-sample phage RPKM values sum to 1 million). Host transcript counts were normalized to the total of host counts *only* (per-sample host RPKM values sum to 1 million). Normalizing host counts to the sum of host but not phage counts accounts for the issue that phage gene expression proportionally displaces host gene expression, with host transcripts becoming a smaller part of the total RNA pool as infection proceeds (S2 Fig), which can complicate determination of whether host transcripts are actually diminished or if they are simply displaced by phage transcripts. Normalizing phage counts to the sum of host *and* phage counts gives an accurate picture of the increase of phage transcription as a fraction of the total pool of transcripts.

### Clustering of genes by expression pattern

Phage genes were clustered by transcript relative abundance patterns using two independent approaches: partitioning around medoids (PAM) and hierarchical clustering. PAM [35] was implemented in R using KL distances [36]. The number of clusters *k* was chosen by finding the lowest value for *k* that nearly maximized the gap statistic; *k* = 5 was chosen as a reasonable number of clusters for both phage and host. Hierarchical clustering was implemented with Cluster 3.0 (http://bonsai.hgc.jp/~mdehoon/software/cluster/software.htm), using uncentered correlation with complete linkage.

### Detection of differential gene expression

Differentially expressed host transcripts (messenger RNAs and antisense RNAs) were identified using the R packages DESeq2 v.1.2.5 [37] and NOISeq v.2.6.0 [38] from the Bioconductor program. Functions and parameters used are listed in S1 Table. Transcript abundances were analyzed at each time point separately, comparing infected to uninfected treatments with light or dark constant and comparing dark to light treatments with infected or uninfected constant. It was not appropriate to compare each time point to a *t* = 0 reference using a repeated measures analysis because the first time point was taken 30 min post-inoculation and already displayed differential expression. Lists of differentially expressed genes (DEGs) were the intersection of lists derived from from DESeq2 and NOISeq, employing a hybrid approach suggested by [39]. DEGs listed were those detected by both NOISeq (NOISeqBIO, probability *q* > 0.95) and DESeq2 (Benjamini–Hochberg adjusted *p*-value < 0.2), with an absolute value of log_2_(fold change) ≥0.4 (infected vs. uninfected) or ≥1.5 (dark vs. light) and total RPKM counts at that time point ≥500 (sense) or ≥100 (antisense).

### Terminology of differential gene expression

We have avoided the terms “up-regulation” and “down-regulation” because we lack evidence of regulatory mechanisms and our data reflect relative but not absolute abundances. We instead favor the terms “enriched” and “diminished” in reference to relative transcript abundance. We have in some cases used the common term “differentially expressed genes” (DEGs); we emphasize that “expression” in this sense refers to transcript abundance only and reflects the net result of transcription minus transcript degradation. We note that the host chromosome is partially degraded during infection (S1 Fig), which complicates interpretation of host gene expression late in infection. We also note that with an MOI of 1, not all host cells would have been infected, which effectively dilutes the detected host response to phage infection. This may account for the observation that even the most differentially expressed host genes did not display transcript fold changes greater than ∼3 in infected versus uninfected.

### Public data

Raw fastq files (80), processed count tables (40), and sample metadata have been submitted to NCBI Gene Expression Omnibus (GEO) with accession number GSE79359. All scripts (R, Python, Perl, and Bash) used in the analysis of this experiment are available on GitHub (https://github.com/cuttlefishh/papers/tree/master/cyanophage-light-dark-transcriptomics).

## Results

### Infection dynamics in light and dark

Infection of *Prochlorococcus* MED4 by cyanophage P-HM2 differed significantly between light and dark conditions. Dynamics of host genome copies, intracellular and extracellular phage genome copies, and pyridine nucleotides (S1 Fig) were measured and reported previously [4]. In light, a burst size of ≥12 was detected after a latent period of ∼8 h, whereas no phage progeny were released in the dark (S1 Fig). Comparing infection in the light versus darkness, degradation of the host chromosome (gDNA) began earlier in the light (1.5 h post-inoculation) than the dark (6.5 h post-inoculation), and by the end of the infection it was nearly complete (86%) in the light compared to 44% in the dark (S1C Fig). Overall, light infection was significantly more robust than dark infection, both in terms of host chromosome degradation, and phage chromosomes synthesized and released as virions. The results of the light and dark infection experiments are summarized in Table 1.

**Table 1:** Results summary.

	Phage gene expression	Host gene expression	Pyridine nucleotides	Productivity of infection
Light early	Early genes: high at 30 min, max at 1.5 h Middle genes: high at 1.5 h, max at 2.5 h	↑ HLIPs, PTOX, PET, Trx, PSI ↓ Cell div, Trx, Transl	Slight increase in NADPH/NADP^+^ and decrease in total phosphorylation state	Host gDNA degraded: 86%, starting at 1.5 h
Light late	Late genes: high at 2.5 h, max at 4.5 h	↑ PSII ↓ FNR, Cell div, Trx, Transl	Strong increase in NADPH/NADP^+^ and decrease in total phosphorylation state	Latent period: 8 h Burst size: ≥12
Dark early	Early genes: max at 30 min, then drop Middle genes: max at 1.5–2.5 h, then drop	Little response	Little change in infected or control	Host gDNA degraded: 44%, starting at 6.5 h
Dark late	Late genes: max at 4.5 h, then drop	↑ Transport, Trx, Transl ↓ FNR, Biosynth, PET, HLIPs, PSI	Total pool more oxidized and less phosphorylated in both infected and control	Latent period: N/A Burst size: N/A

Events are listed for light and dark experiments, divided into early (0–2 h post-inoculation) and late (2–9 h post-inoculation) periods. Abbreviations: Biosynth, biosynthesis; Cell div, cell division; FNR, ferredoxin–NADP^+^ reductase; HLIPs, high light inducible proteins; PET, photosynthetic electron transport; PSII, photosystem II; PTOX, plastoquinol terminal oxidase; Transl, protein translation; Trx, transcription; N/A, not applicable.

### Phage gene expression in light and dark during infection

Differences in overall phage transcription between light and dark were consistent with the differences in infection dynamics. In light infection, phage transcripts (as a percentage of total phage + host transcripts) increased from 3% 0.5 h post-inoculation to >65% at 8.5 h (S2 Fig). Conversely, phage transcripts never constituted more than 2% of the total at any point in dark infection (S2 Fig).

**Fig 1:**
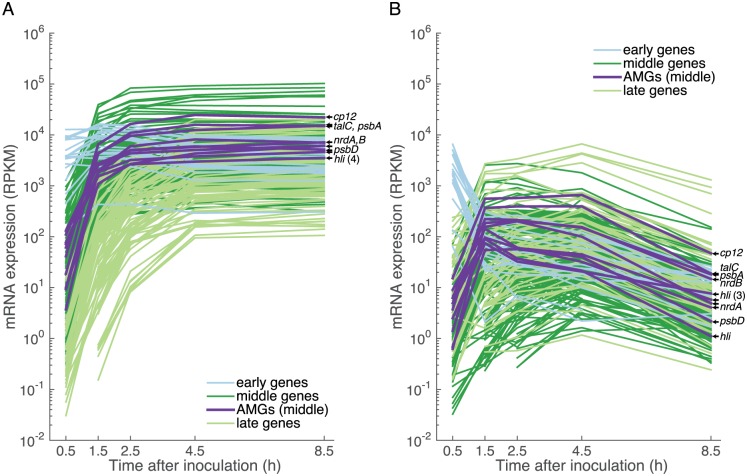
Phage gene expression in early, middle, and late temporal clusters during light and dark infection. Relative expression of phage mRNA is in units of reads per kbp gene length per million (RPKM) of host and phage reads (see methods). Phage auxiliary metabolic genes (AMGs) are labeled: *cp12*, Calvin cycle inhibitor CP12; *talC*, transaldolase; *psbA* and *psbD*, photosystem II D1 and D2 proteins; *nrdA* and *nrdB*, ribonucleotide reductase alpha and beta subunits; and *hli*, high-light inducible proteins.

Clustering phage genes by timing of expression revealed few light-dependent differences. While there were three expression clusters—early, middle, and late (Fig 1), as seen in other T4-like cyanophage transcriptomes [22, 40]—the majority (93%) of phage genes were expressed in the same time cluster in light and dark (S1 Table), and their timing of peak expression was similar under both conditions: early gene expression peaked at 0.5 h, middle at 2.5 h, and late at 4.5 h (Fig 1). Timing of gene expression among T4-like cyanophages appears to be conserved, as 95% of genes shared between cyanophage P-HM2 used here and cyanophage P-SSM2 [40] were expressed in the same time cluster.

Average genome-wide phage gene expression levels were 3x greater in the light relative to the dark at the beginning of infection (0.5 h), then diverged dramatically at intermediate time points reaching 35x greater at 2.5 h; at the end of the infection average expression levels in the light were 465x those in the dark. While middle genes were the most highly expressed across the phage transcriptome in the light, in darkness their expression dropped after 1.5 h and late gene expression became the most abundant. Thus, based on expression levels, middle genes were the most responsive to light.

Phage auxiliary metabolic genes (AMGs) were highly expressed in the light, relative to the rest of the phage transcriptome and relative to AMG expression in the dark; AMGs were expressed exclusively in the middle transcription cluster (Fig 1 and S2 Table). The two AMGs involved in the pentose phosphate pathway, *cp12* (Calvin cycle inhibitor CP12) and *talC* (transaldolase), were among the most highly expressed phage genes in the light (Fig 1). While we previously showed that phage *psbA*, *cp12*, *talC*, *nrdA*, and *nrdB* were expressed concurrently with known T4-like phage early/middle genes (*g61*, *g43*) [4], we could not place the expression levels of those genes in context with the rest of the phage transcriptome. Here we show the high expression of phage AMGs involved in PET, the pentose phosphate pathway, and ribonucleotide reductase—relative to the rest of the phage transcriptome, and in light relative to dark—thereby indicating that they are poised to influence host light-driven metabolism.

### Host gene expression in light and dark during infection

**Fig 2:**
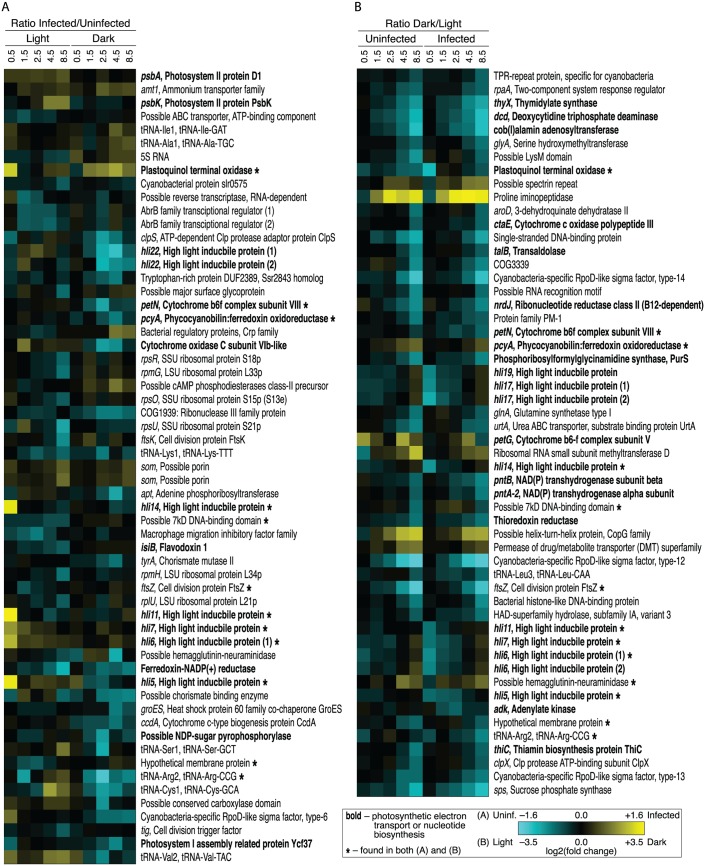
Heat maps of differentially expressed genes in light and dark infections (infected:uninfected ratio) and in uninfected and infected treatments (dark:light ratio). Genes are listed in order on the MED4 chromosome. Bold gene labels indicate involvement in photosynthetic electron transport or nucleotide biosynthesis, and asterisks indicate genes found in both heat maps. The color gradient indicates gene transcripts enriched (yellow) or diminished (cyan) as follows: (A) in infected relative to uninfected treatment in one or more light or dark time point or (B) in dark relative to light in one or more uninfected or infected time point (note the different scales).

From the perspective of the *Prochlorococcus* host, our experimental design involved four conditions: (1) cells maintained in constant light (control); (2) cells shifted to dark; (3) cells remaining in the light and infected with phage; and (4) cells infected and immediately shifted to dark. Host transcript counts were normalized to the total number of host transcript counts, and reported fold changes are between RPKM values in dark relative to light or infected relative to uninfected (see methods). As might be expected, the effects on host gene expression resulting from light deprivation were much more pronounced and sweeping (Fig 2B and S4 Table) than from phage infection alone in the light or in darkness (Fig 2A and S3 Table). Stated another way, light deprivation induced a similar transcriptional response in the host whether or not it was infected, with cell division (*ftsZ*) and sigma factor (*rpoD*) transcripts among the most diminished, and proline iminopeptidase and transcriptional regulation (*copG*) transcripts among the most enriched (Fig 2B).

Light deprivation did not overwhelm the effect of phage infection in two notable cases: high-light inducible genes (*hli5,6,7,11,14,17,19*) and plastoquinol terminal oxidase (PTOX) were enriched in light (depleted in dark) much more so in infected than uninfected cells. As discussed below, PTOX and *hli* genes appear to be key elements of the induced host response to the combined effects of light and phage.

#### Host genes differentially expressed in light infection

**Fig 3:**
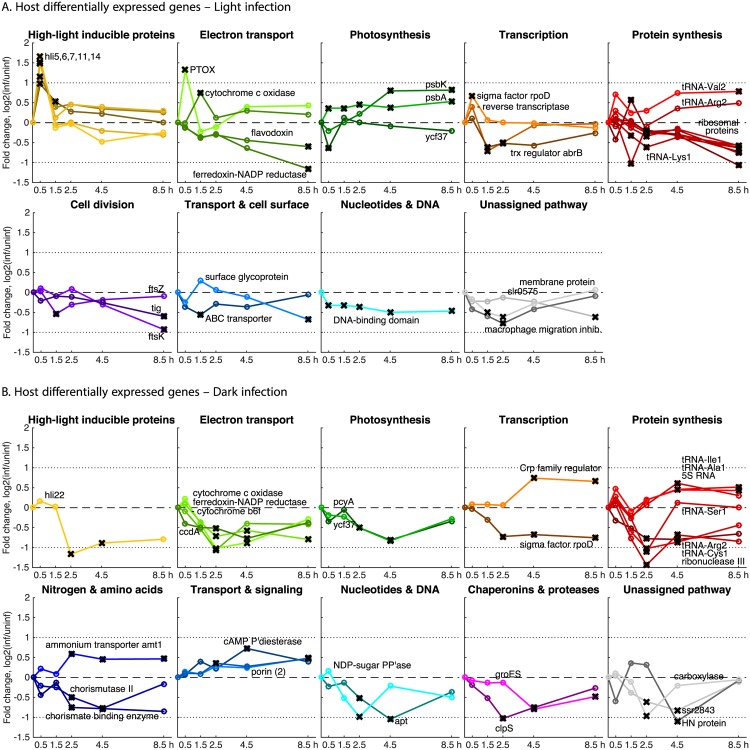
Host differentially expressed genes (DEGs) following infection in the light and dark, organized by pathway. Genes shown were differentially expressed at one or more time point, marked with a black ‘X’ if NOISeq probability *q* > 0.95, and match those in S3 Table. Line color shading is proportional to relative transcript abundance at 0.5 h post-inoculation. Two-fold changes up and down are indicated by dotted lines, and zero fold change is indicated by a dashed line.

Most (90%) of host genes that exhibited differential expression during infection did so either in the light or the dark, but not both (Fig 2A). Among host genes with increased relative expression in light (but not in dark) infection (Figs 3A and 2A and S3 Table) were those encoding components of PET—*hli* genes, *psbA* and *psbK* and cytochrome *c* oxidase (COX)—suggesting a requirement for maintenance of electron flow in the thylakoid membrane. Host sigma factor RpoD transcripts were enriched in light infection but diminished in dark infection. The possible roles of RpoD and other transcription factors during light and dark infection are addressed in the discussion.

The finding that host high-light inducible (*hli)* genes were induced by infection only in the light suggests that this response is not a general stress response to phage infection. To gain insight into this infection-induced response, we compared expression of the 18 unique *hli* genes in *Prochlorococcus* MED4 across various conditions in published transcriptomics studies (S7 Table). The MED4 response to cyanophage P-HM2 infection in light had the same five enriched transcripts as the response to cyanophage P-SSP7 infection [21], and also to high-light stress [41] and iron stress [42], and coincided with a diel expression cluster that peaked around 1:00–2:00 am [43]. Genes transcribed in the early morning in MED4 had corresponding proteins present by sunrise, 2–8 hours later [44]. Iron is a critical metal cofactor for photosystems. Therefore it appears the set of *hli* genes differentially expressed in all these experiments has an important role in responding to the oxidative and other stresses incurred by photosynthesis.

That host *psbA* was enriched in light infection but not dark infection is consistent with a model in which phage-encoded PsbA protein is inserted into photodamaged photosystems to prevent photoinhibition [15, 18, 45–47]. As with the host *hli* response, the host *psbA* and *psbK* response was contingent on both infection and light, indicating that photoinhibition and oxidative stress are exacerbated by phage infection in the presence of light.

Host transcripts that were diminished in light infection included photosystem I assembly protein Ycf37 and ferredoxin–NADP^+^ reductase (FNR)—which were also diminished in dark (discussed below)—and translation-related genes including multiple tRNAs and ribosomal proteins, consistent with the significant changes in protein translation that must occur in the shift from host to phage protein synthesis. Cell division transcripts *ftsZ*, *ftsK*, and *tig* were also diminished in light infection. It is notable that *ftsZ* transcripts were also diminished in all dark treatments, suggesting similar inhibitory effects of darkness and infection on cell division.

#### Host genes differentially expressed in dark infection

Few host genes were differentially expressed early in dark infection, consistent with the low efficiency of phage infection (S1 Fig) and gene expression (Fig 1), as expected in an infection slowed by the removal of photosynthetic energy. Later in infection, however, a significant host transcriptional response was observed (Figs 3B, 2A and S3 Table), though largely distinct from the differentially expressed genes observed in light infection. Transcripts enriched only in dark infection included those encoding transport functions (*amt1* for ammonium transporter, several porins), transcription (Crp family), and translation (tRNAs and 5S RNA).

Diminished transcripts included *hli22* (not among the enriched *hli* transcripts in light infection), PET (cytochrome *b*_6_ *f*, COX, and FNR), pigment biosynthesis (*pcyA*), LPS biosynthesis, proteolysis (*clpS*), nuclease (RNase III), transcription (*rpoD*), and translation (tRNAs). The increase in transcripts of genes encoding several porins and an ammonium transporter (*amt1*) suggests that ammonium and other nutrients may have been limiting to host or phage during dark infection. The decrease in transcripts of genes encoding cytochrome *b*_6_ *f* and COX is notable in that these complexes have been proposed to help maintain electron flow under oxidative stress [48] or phage infection [30]; in fact, we observed COX transcripts to be enriched in light infection.

#### Host genes differentially expressed in both light and dark infection

Only five host genes, three of which are implicated in PET, were differentially expressed in both light and dark infection using the defined threshold criteria (Fig 2A and S3 Table). Two of the five were enriched in light infection but diminished in dark infection: COX subunit VIb-like and RpoD-like sigma factor. The other three were diminished in both light and dark infection: FNR, tRNA-Arg2, and photosystem I assembly protein Ycf37. A sixth transcript, for PTOX, was significantly enriched early in light infection. While PTOX was not identified as differentially expressed in dark infection, it did display an increasing trend later in dark infection (Fig 2A). This suggests that the PTOX response, unlike that of other PET genes, is induced in dark infection as well as light.

## Discussion

### Refining the model of photosynthetic electron transport during cyanophage infection

**Fig 4:**
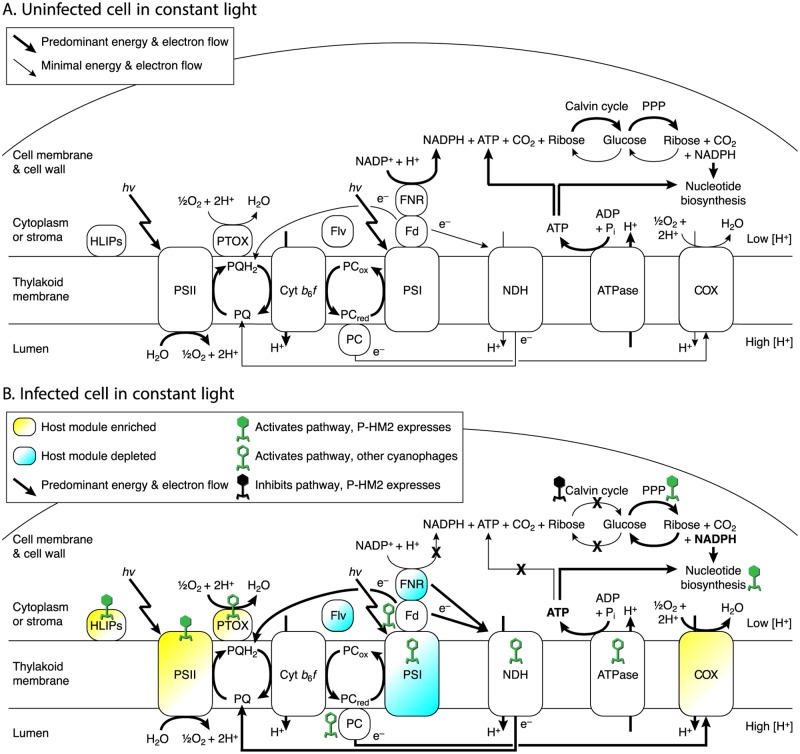
Schematic of proposed flow of electrons, energy, and carbon in cyanophage-infected cyanobacteria in the light. In the dark (not pictured), PET and phage replication are stunted because the cell is not receiving light energy. In the light, however, energy (*hν*) excites electrons in water to travel through the photosynthetic electron chain, with the terminal electron acceptor being NADP^+^ via ferredoxin–NADP^+^ reductase (FNR) in uninfected cells (A) and oxygen via plastoquinol terminal oxidase (PTOX) or cytochrome *c* oxidase (COX) in infected cells (B). Under phage infection, host FNR transcripts are diminished, and therefore the proposed terminal electron acceptor is not NADP^+^. Rather, cyclic electron flow goes through NAD(P)H dehydrogenase (NDH) or direct to plastoquinone, with PTOX serving as the terminal electron acceptor. Cyanophage P-HM2 genes (filled viruses) are transcribed for high-light inducible proteins (HLIPs), photosystem II (PSII), the pentose phosphate pathway (PPP), and nucleotide biosynthesis; additional cyanophage genes not in P-HM2 (unfilled viruses) exist for modules along this metabolic network. NADPH is proposed to be generated from pre-existing reduced carbon (glucose) via the PPP. Any NADPH produced by FNR would be designated for the Calvin cycle by docking of FNR with GAPDH and therefore unproductive because of Calvin cycle inhibition by phage CP12. In this model of light-driven phage infection, therefore, the main source of energy (ATP) is cyclic photosynthesis and of reducing equivalents (NADPH) is the PPP, with ATP and NADPH consumed in the production of nucleotides to replicate the phage genome. Other abbreviations: PQ/PQH2, plastoquinone/plastoquinol (oxidized/reduced); Cyt *b*_6_ *f*, cytochrome *b*_6_ *f*, PC_ox_/PC_red_, plastocyanin (oxidized/reduced); PSI, photosystem I; Fd, ferredoxin; ATPase, F_1_F_0_-ATP synthase.

A model for changes in electron flux through photosynthetic electron transport during cyanophage infection has been developed over the last decade [4, 6, 15, 18, 21, 26, 29, 30]. Fig 4 summarizes this synthesis and incorporates observed phage and host gene expression patterns from the present study. In an uninfected cell (Fig 4A) in the light, photosynthetic energy is used to split water, whose electrons flow through the electron transport chain, generating a proton gradient to power ATP synthesis, and the electrons are passed to NADP^+^ to make NADPH for carbon fixation. Under this model, electron transport in uninfected cells is predominantly linear. In an infected cell (Fig 4B) in the light, oxygen rather than NADP^+^ is the main terminal electron acceptor. Mounting evidence supports the hypothesis that cyclic photophosphorylation around photosystem I is the predominant mode of electron transport in phage-infected hosts [20, 27–29], preferentially producing ATP over NADPH. NADPH is not consumed by carbon fixation, which is inhibited under phage infection [26], and instead the pentose phosphate pathway is the primary source of NADPH [4]. The sink of ATP and NADPH is proposed to be cyanophage nucleotide biosynthesis. The additional evidence here in support of the stated model is as follows.

There are several lines of evidence against NADP^+^ and for oxygen as the terminal electron acceptor during infection. Transcripts of ferredoxin–NADP^+^ reductase (FNR) were the most diminished of any host gene in light infection. In linear PET, NADP^+^ receives electrons from ferredoxin (Fd) via FNR, and the resulting NADPH is used by glyceraldehyde-3-phosphate dehydrogenase (GAPDH) in the first step of the Calvin cycle. The large decrease in host FNR transcripts during infection points to diminished electron flow through this oxidoreductase. We note that FNR would normally pass NADPH directly to GAPDH via protein–protein docking, meaning that any NADPH produced by FNR would be earmarked for carbon fixation. Docking of enzymes to pass substrates from one enzyme to the next in a pathway is an increasingly recognized feature of bacterial metabolism [49]. FNR is a soluble complex [50], able to bind to ferredoxin on the thylakoid membrane and move through the cytosol. Notably, in pea leaf chloroplasts [51] and diatom plastids [52], FNR binds to glyceraldehyde-3-phosphate dehydrogenase (GAPDH), consistent with NADPH produced by FNR being passed directly to GAPDH. Adding to this idea, the phage gene for CP12, which regulates the Calvin cycle in cyanobacteria [25], was among the most highly expressed phage genes in the light, suggesting that GAPDH in the Calvin cycle is inhibited, consistent with [26]. A phage ‘strategy’ to down-regulate FNR to produce less NADPH destined for carbon fixation would also benefit from inhibiting carbon fixation.

Host PTOX and COX transcripts were both enriched in light infection, pointing to these as likely terminal oxidases, transferring electrons to the terminal acceptor oxygen (Fig 4). PTOX is thought to function as a safety valve for PET in cyanobacteria, passing electrons from the PQ pool (plastoquinol, reduced; plastoquinone, oxidized) to oxygen when PQ becomes overly reduced [53, 54]. Such over-reduction can occur under high light [55], and when photosystem I [56] and carbon fixation [57] are unable to accept electron flow. *Prochlorococcus* PTOX gene expression is induced by high light [41], iron starvation [42], and inorganic carbon limitation [57]. *Synechococcus* WH8102 PTOX, a distant homolog of those in *Prochlorococcus* and cyanophage, can reduce oxygen with electrons from plastoquinone [53], consistent with cyclic electron flow around PSII, eliminating the need for PSI and FNR-mediated NADPH production [30]. COX also uses oxygen as its terminal acceptor [58] and can accept electrons from PQ when PSI is absent [56]. PSI may have been down-regulated in light infection, as expression of *ycf37*, encoding photosystem I assembly protein Ycf37 [59], was diminished. The increase in host COX transcripts in light infection supports a recent hypothesis [30] for the role of this complex in accepting electrons from the plastocyanin (PC) pool during infection (Fig 4).

The expression dynamics of both phage and host genes support the hypothesis that the PPP is the major source of NAPDH during infection. Phage genes for the PPP enzyme transaldolase (*talC*) and the Calvin cycle inhibitor CP12 (*cp12*) highly expressed during light infection (S4D Fig). Early in light infection, host genes for the key Calvin cycle enzymes glyceraldehyde-3-phosphate dehydrogenase (*gap2*) and phosphoribulokinase (*prkB*) are diminished (S4 Fig) while the host gene for CP12 (*cp12*) is enriched (S4C Fig). The Calvin cycle and pentose phosphate pathway (PPP) run in counter directions and cannot productively run at the same time (S4E Fig). In the light, therefore, the down-regulation of the Calvin cycle and up-regulation of the PPP inferred from both host and phage transcription indicates the PPP is active and is poised to oxidize carbon stores to produce NADPH and possibly pentose sugars for phage nucleotide biosynthesis.

NADPH produced by the PPP in infected cells would be available for nucleotide biosynthesis, in particular ribonucleotide reductase (RNR) activity. RNR genes (*nrdA* and *nrdB*) were among the highly expressed phage middle cluster. If NADPH produced by the PPP is consumed by dNTP biosynthesis, one might expect the NADPH/NADP^+^ ratio to remain steady, not increase [30]. However, the stoichiometry of dNTPs required for phage replication, plus the high expression of phage RNR, imply an increase in RNR activity during infection, and thus increased NADPH consumption. Under these assumptions, an increase in the NADPH/NADP^+^ ratio can only mean an increase in NADPH production.

### The role of light in cyanophage infection

The comparison of infection in light versus dark provides insight into light-dependent regulation of cyanophage infection. The transcriptional pattern of *rpoD* (host RpoD-like sigma factor 70) in particular is notable. Host sigma factors are required for T4-like phage expression of middle and late genes [60], and the host sigma factor encoded by *rpoD* is a likely candidate for this role. Because *rpoD* transcription is enriched in the light and depleted in the dark, this could be a primary mechanism for regulation of phage development by light. Additionally, transcripts of *copG*, for the transcription factor CopG, were enriched in the dark in both infected and uninfected treatments. CopG is a transcriptional repressor that hinders binding of RNA polymerase to promoter regions [61], which could contribute to additional repression of phage transcription in the dark. However, under lab-controlled diel conditions, CopG from this same strain (MED4) was maximally expressed in the daytime (see CyCOG 3686 at proportal.mit.edu and [43]), and therefore its transcription appears to react differently to gradual versus dramatic shifts between light and dark.

It is also notable that the phage transcriptional program was able to proceed in the same order in the dark as in the light, albeit with much lower efficiency. This could be due to low-level infection by wild-type phage using residual host energy stores, following a defined program dependent on available energy but independent of light. This could also be explained by a small population of mutant phage whose transcription is not repressed in the dark, although we have no evidence of direct dark-dependent repression of phage transcription in our study. Future work to try to isolate any such mutants, and to determine the effects of adding back light to dark-inoculated cultures, including over a 24-h light–dark cycle, would be fruitful.

## Conclusion

The results presented here provide insight into the role of light in cyanophage–host transcription and cyanophage takeover of host metabolism. The transcriptional patterns, particularly of the host genes, point to key steps in host phototrophic metabolism toward a metabolic landscape that supports phage reproduction. In particular, it appears that one reason phage infection is enhanced by light is that phage can direct PET to favor cyclic photophosphorylation and ATP production, using oxidases as terminal electron acceptors. Depletion of host FNR transcripts during infection in the light, and putative protein–protein docking between FNR and GAPDH in the Calvin cycle, point towards minimal NADPH production by PET. Phage augmentation of the pentose phosphate pathway provides a more direct mechanism for NADPH production, where NADPH is not destined for a counter-productive pathway (carbon fixation), but rather both ATP and NADPH are available for nucleotide synthesis for phage genome replication. Support of this model will require biochemical understanding of its individual elements, such as protein complexes with FNR and CP12, and changes in the flow of electrons as infection proceeds. Investigations into the intrinsic role of light will eventually need to incorporate the host’s light–dark cycle and cell cycle [62], which are confounding but ultimately essential aspects of this phage–host metabolic parasitism in the wild.

## Supporting Information

S1 Fig Infection dynamics and pyridine nucleotide ratios in light and dark experiments. Data are replotted from [4], with the difference that time after inoculation is 0.5 h later than previously reported to better reflect the time to process samples. (A–C) Genome copies were quantified by qPCR of *g20* (phage) and *rnpB* (host). Error bars represent standard deviations of two biological and three technical replicates. (D–F) Pyridine nucleotide data are ratios of NADPH/NADP^+^ (reduced/oxidized forms of NADPH), NADP(H)/NAD(H) [phosporylated/unphosphorylated forms of NADP(H)], and ratios of infected to uninfected values of those two ratios. Error bars represent standard deviations of two biological and two technical replicates.
(PDF)

S2 Fig Fraction of host and phage reads in light and dark experiments. Note that the y-axis is linear in the light plot (A) and logarithmic in the dark plot (B).
(PDF)

S3 Fig Genomic context of differential gene expression and sense/antisense ratio in light and dark infection. Average fold change (log2(infected/uninfected)) for early (blue; 0.5, 1.5 h) and late (red; 2.5, 4.5, 8.5 h) portions of (A) light and (B) dark infection experiments. Averages across all treatments and time points of (C) sense RNA (mRNA, green) and antisense RNA (asRNA, pink) levels (RPKM-normalized), and (D) ratio of sense to antisense RNA (black). Each gene is a point, with genes numbered from 1 to 2015 along the MED4 chromosome. Genomic islands ISL1–ISL5 in MED4 (Coleman et al., Genomic islands and the ecology and evolution of *Prochlorococcus*. Science. 2006;311(5):1768–1770) are shown for reference (cyan).
(PDF)

S4 Fig Expression dynamics of host Calvin cycle and pentose phosphate pathway genes, including genes shared between the two pathways, in infection in light and dark. Note the y-scale in host PPP genes (C) spans a wider range than in host Calvin cycle genes (A) and host shared PPP/Calvin cycle genes (B). Also shown is (D) phage PPP gene expression in light and dark. (E) Pathway diagram shown for reference; key genes are bolded: *cp12* (CP12; host and phage), *prkB* (PRK; host), *gap2* (GAPDH; host), and *talB* (TalB, host)/*talC* (TalC, phage).
(PDF)

S1 Table Functions and parameters used for detection of differential host gene expression. The packages used were DESeq2 (v.1.2.5) and NOISeq (v.2.2.1) from the Bioconductor program in R.
(PDF)

S2 Table Phage gene expression clusters during light and dark infection. Genes were clustered by RPKM-normalized transcript levels into five clusters (A–E), designated as early (A), middle (B, C), and late (D, E), based on timing and pattern of gene expression. Listed are NCBI locus tags, gene names (where available), expression clusters in light and dark, and whether those clusters are the same.
(PDF)

S3 Table Differential transcript abundance of host genes in light and dark in infected relative to uninfected cultures. RPKM-normalized counts and log2(fold change) are given for infected relative to uninfected duplicates (NOISeq) in light (Part A) and dark (Part B). NCBI locus tags for *Prochlorococcus* MED4 are provided. DEGs listed are those detected by both NOISeq and DESeq2 (S1 Table), with an absolute value of log2(fold change) ≥0.4 and total counts at that time point ≥500. Hypothetical proteins are excluded.
(PDF)

S4 Table Differential transcript abundance of host genes in uninfected and infected cultures in the dark relative to the light. RPKM-normalized counts and log2(fold change) are given for dark relative to light duplicates (NOISeq) in uninfected (Part A) and infected (Part B). NCBI locus tags for *Prochlorococcus* MED4 are provided. DEGs listed are those detected by both NOISeq and DESeq2 (S1 Table), with an absolute value of log2(fold change) ≥1.5 and total counts at that time point ≥500. Hypothetical proteins are excluded.
(PDF)

S5 Table Differential transcript abundance of host antisense RNAs in light and dark in infected relative to uninfected cultures. RPKM-normalized counts and log2(fold change) are given for infected relative to uninfected duplicates (NOISeq) in light (Part A) and dark (Part B). NCBI locus tags for *Prochlorococcus* MED4 are provided. DEGs listed are those detected by both NOISeq and DESeq2 (S1 Table), with an absolute value of log2(fold change) ≥0.4 and total counts at that time point ≥100. Hypothetical proteins are excluded in Part B.
(PDF)

S6 Table Host genes with antisense/sense ratios averaged across all samples greater than 10. NCBI locus tags for *Prochlorococcus* MED4 are provided. Hypothetical proteins are included.
(PDF)

S7 Table High-light inducible gene expression in *Prochlorococcus* MED4. Previously conducted experiments have examined the transcriptome of *Prochlorococcus* MED4 under a diel (light–dark) cycle [43], high-light stress [41], low dissolved ammonium and urea (Tolonen et al., Global gene expression of *Prochlorococcus* ecotypes in response to changes in nitrogen availability. Mol Syst Biol. 2006;2:53), low dissolved phosphate (Martiny et al., Phosphate acquisition genes in *Prochlorococcus* ecotypes: Evidence for genome-wide adaptation. Proc Natl Acad Sci USA. 2006;103(33):12552–12557), low dissolved iron [42], low dissolved inorganic carbon [57], and infection by podovirus P-SSP7 in continuous light [21]. Experiments presented here examined the transcriptome of *Prochlorococcus* MED4 under infection by myovirus P-HM2 in continuous light or following a shift to dark. Plus and minus signs indicate transcripts enriched or diminished in the respective treatment. Brackets around signs indicate transcripts detected as enriched or diminished by only one of NOISeq or DESeq2.
(PDF)
