# Supplementary figures and images for: Gene Expression Patterns during Light and Dark Infection of Prochlorococcus by Cyanophage
Source: PLoS One. 2016 Oct 27;11(10):e0165375. doi: 10.1371/journal.pone.0165375 (PMC5082946; doi:10.1371/journal.pone.0165375)

**S1 Fig**

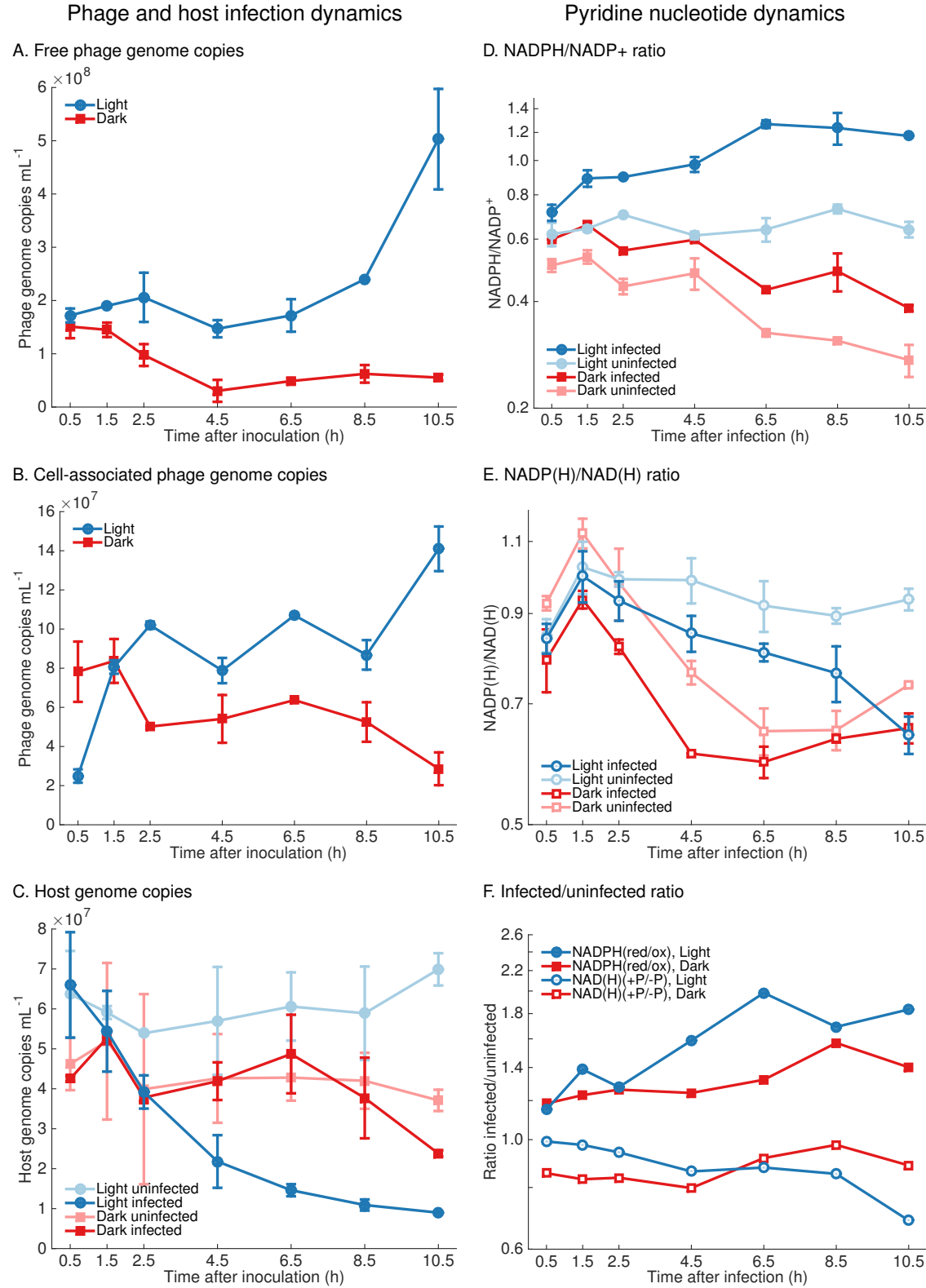

Supplement: S1 Fig — Data are replotted from [4], with the difference that time after inoculation is 0.5 h later than previously reported to better reflect the time to process samples. (A–C) Genome copies were quantified by qPCR of g20 (phage) and rnpB (host). Error bars represent standard deviations of two biological and three technical replicates. (D–F) Pyridine nucleotide data are ratios of NADPH/NADP+ (reduced/oxidized forms of NADPH), NADP(H)/NAD(H) [phosporylated/unphosphorylated forms of NADP(H)], and ratios of infected to uninfected values of those two ratios. Error bars represent standard deviations of two biological and two technical replicates. (PDF) [file pone.0165375.s001.pdf]

**S2 Fig**

**A. Read distribution – Light infection**

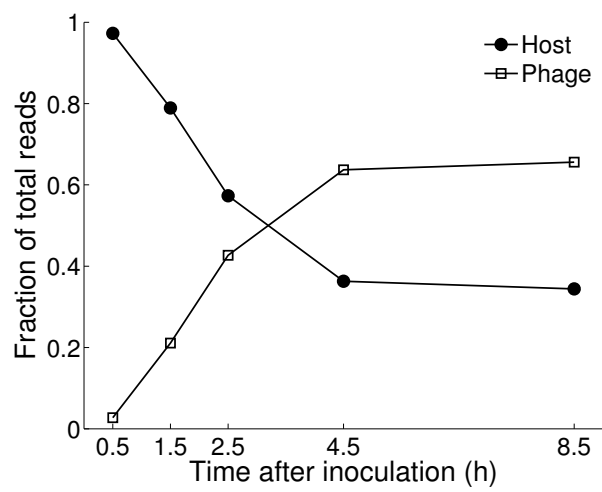

**B. Read distribution – Dark infection**

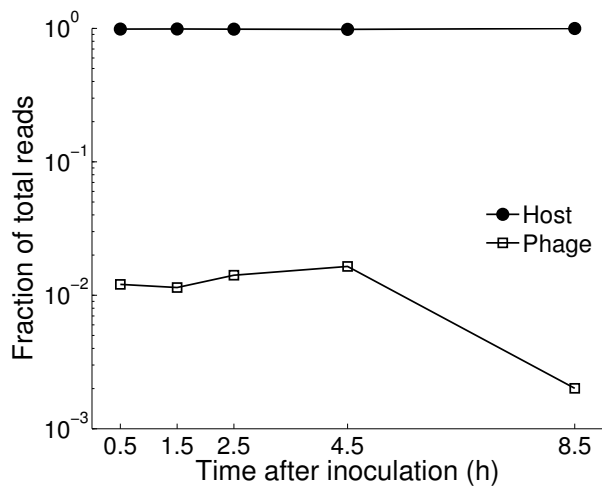

Supplement: S2 Fig — Note that the y-axis is linear in the light plot (A) and logarithmic in the dark plot (B). (PDF) [file pone.0165375.s002.pdf]

S3 Fig

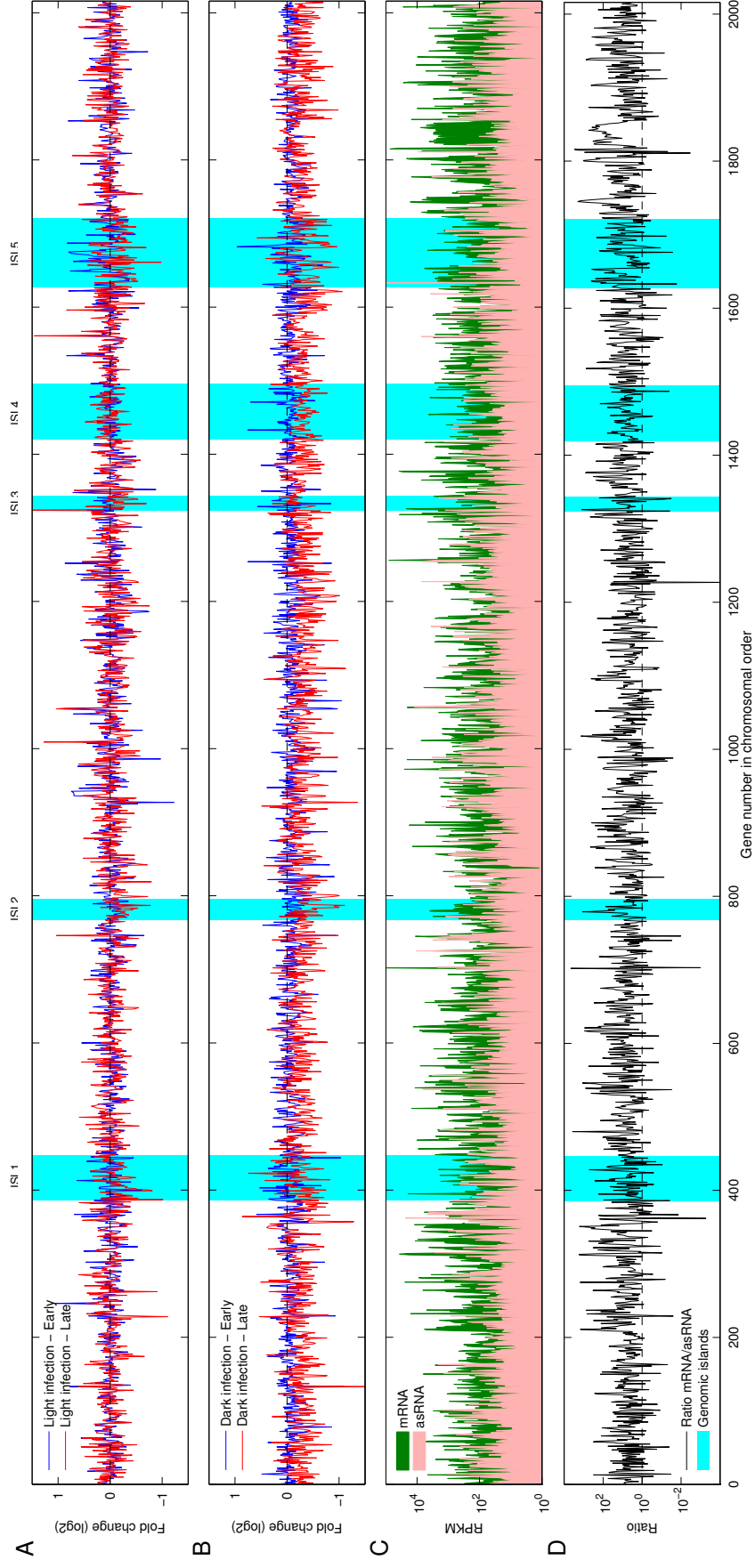

Supplement: S3 Fig — Average fold change (log2(infected/uninfected)) for early (blue; 0.5, 1.5 h) and late (red; 2.5, 4.5, 8.5 h) portions of (A) light and (B) dark infection experiments. Averages across all treatments and time points of (C) sense RNA (mRNA, green) and antisense RNA (asRNA, pink) levels (RPKM-normalized), and (D) ratio of sense to antisense RNA (black). Each gene is a point, with genes numbered from 1 to 2015 along the MED4 chromosome. Genomic islands ISL1–ISL5 in MED4 (Coleman et al., Genomic islands and the ecology and evolution of Prochlorococcus. Science. 2006;311(5):1768–1770) are shown for reference (cyan). (PDF) [file pone.0165375.s003.pdf]

**S4 Fig**

### A. Host – Calvin cycle

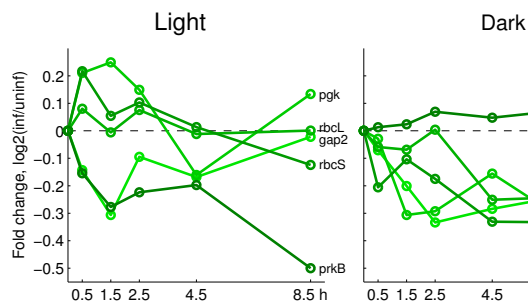

### B. Host – Shared PPP/Calvin cycle

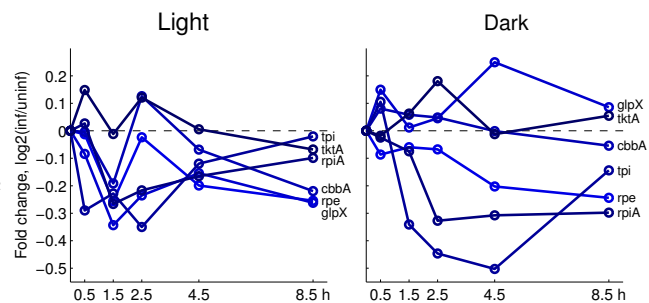

### C. Host – PPP

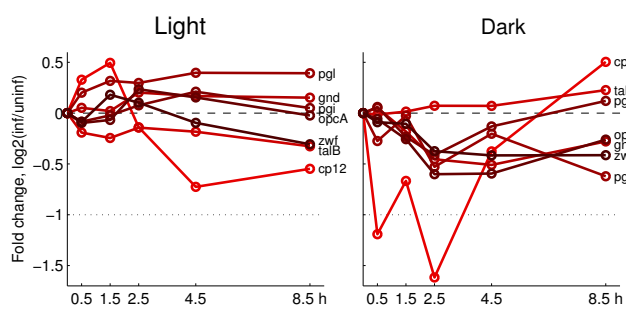

#### D. Phage – PPP

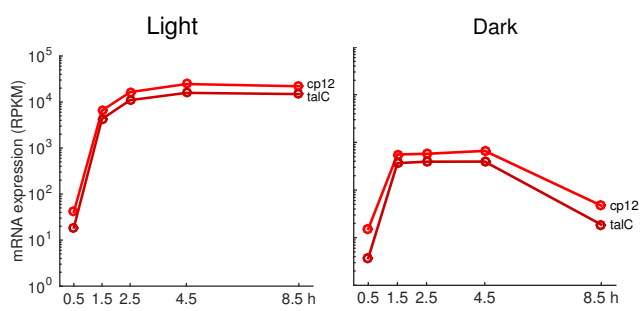

### E. Pathway diagram

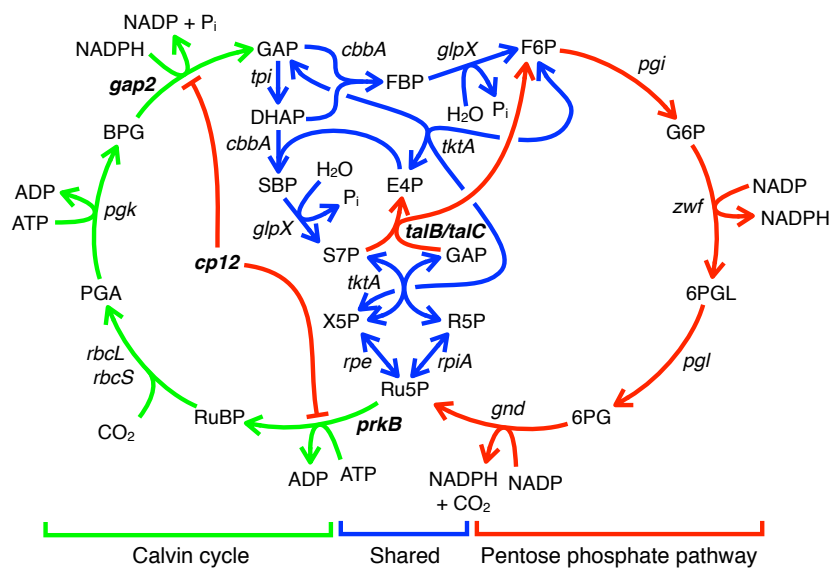

Supplement: S4 Fig — Note the y-scale in host PPP genes (C) spans a wider range than in host Calvin cycle genes (A) and host shared PPP/Calvin cycle genes (B). Also shown is (D) phage PPP gene expression in light and dark. (E) Pathway diagram shown for reference; key genes are bolded: cp12 (CP12; host and phage), prkB (PRK; host), gap2 (GAPDH; host), and talB (TalB, host)/talC (TalC, phage). (PDF) [file pone.0165375.s004.pdf]
