# Supplementary material for: Gene Expression Patterns during Light and Dark Infection of Prochlorococcus by Cyanophage
Source: PLoS One. 2016 Oct 27;11(10):e0165375. doi: 10.1371/journal.pone.0165375 (PMC5082946; doi:10.1371/journal.pone.0165375)
Supplement: S1 Table — The packages used were DESeq2 (v.1.2.5) and NOISeq (v.2.2.1) from the Bioconductor program in R. (PDF) [file pone.0165375.s005.pdf]

S1 Table

	DESeq2	NOISeq
Prefiltering count data (NOISeq function used for both)	`filtered.data`<br>`method=1`<br>`cv.cutoff=10`<br>`cpm=5`	`filtered.data`<br>`method=1`<br>`cv.cutoff=10`<br>`cpm=5`
Calculating differential expression statistics	`estimateSizeFactors`<br>`estimateDispersions`<br>`nbinomWaldTest`	`noiseqbio`<br>`k=0.5`<br>`norm=rpkm`<br>`lc=1`<br>`r=50`<br>`adj=1.5`<br>`nclust=15`<br>`a0per=0.9`<br>`random.seed=12345`<br>`filter=0`
Reporting differentially expressed genes	`results`<br>`independentFiltering=FALSE`<br>`padj<0.2`<br>`abs(log2FoldChange)>0.5`	`degenes`<br>`q=0.95`
