# Supplementary material for: Gene Expression Patterns during Light and Dark Infection of Prochlorococcus by Cyanophage
Source: PLoS One. 2016 Oct 27;11(10):e0165375. doi: 10.1371/journal.pone.0165375 (PMC5082946; doi:10.1371/journal.pone.0165375)
Supplement: S2 Table — Genes were clustered by RPKM-normalized transcript levels into five clusters (A–E), designated as early (A), middle (B, C), and late (D, E), based on timing and pattern of gene expression. Listed are NCBI locus tags, gene names (where available), expression clusters in light and dark, and whether those clusters are the same. (PDF) [file pone.0165375.s006.pdf]

S2 Table

Locus tag	Gene	Light	Dark	Same?
PHM2_001	*g59*	Mid (B)	Mid (C)	+ (–)
PHM2_002		Mid (B)	Mid (C)	+ (–)
PHM2_003		Mid (B)	Mid (C)	+ (–)
PHM2_004		Mid (C)	Mid (C)	+ (+)
PHM2_005		Mid (C)	Mid (C)	+ (+)
PHM2_006		Mid (C)	Mid (C)	+ (+)
PHM2_007		Mid (C)	Mid (C)	+ (+)
PHM2_008		Mid (C)	Mid (C)	+ (+)
PHM2_009	*denV*	Mid (C)	Mid (C)	+ (+)
PHM2_010		Mid (C)	Mid (C)	+ (+)
PHM2_011		Mid (C)	Mid (C)	+ (+)
PHM2_012		Mid (C)	Mid (C)	+ (+)
PHM2_013	*g32*	Mid (B)	Mid (C)	+ (–)
PHM2_014		Late (E)	Late (E)	+ (+)
PHM2_015	*g53*	Late (E)	Late (E)	+ (+)
PHM2_016	*g48*	Late (E)	Late (E)	+ (+)
PHM2_017	*g2*	Late (E)	Late (E)	+ (+)
PHM2_018	*g4*	Late (E)	Late (E)	+ (+)
PHM2_019		Late (E)	Late (E)	+ (+)
PHM2_020	*g26*	Late (E)	Late (E)	+ (+)
PHM2_021	*g51*	Late (E)	Late (E)	+ (+)
PHM2_022		Late (E)	Late (E)	+ (+)
PHM2_023		Late (E)	Late (E)	+ (+)
PHM2_024		Late (E)	Late (E)	+ (+)
PHM2_025	*g5*	Late (D)	Late (E)	+ (–)
PHM2_026	*g5*	Late (D)	Mid (C)	– (–)
PHM2_027		Late (D)	Late (E)	+ (–)
PHM2_028		Mid (C)	Mid (C)	+ (+)
PHM2_029		Mid (B)	Mid (C)	+ (–)
PHM2_030		Mid (B)	Mid (C)	+ (–)
PHM2_031	*cpeT*	Mid (B)	Mid (C)	+ (–)
PHM2_032		Late (E)	Late (D)	+ (–)
PHM2_033		Late (E)	Late (E)	+ (+)
PHM2_034		Late (E)	Late (E)	+ (+)
PHM2_035		Late (E)	Late (E)	+ (+)
PHM2_036		Late (E)	Late (E)	+ (+)
PHM2_037		Late (D)	Late (D)	+ (+)
PHM2_038		Mid (C)	Mid (C)	+ (+)
PHM2_039		Mid (C)	Mid (C)	+ (+)
PHM2_040		Mid (C)	Mid (C)	+ (+)
PHM2_041	*denV*	Mid (C)	Mid (C)	+ (+)
PHM2_042		Mid (C)	Mid (C)	+ (+)
PHM2_043	*dam*	Mid (C)	Mid (C)	+ (+)
PHM2_044		Mid (B)	Mid (B)	+ (+)
PHM2_045		Mid (B)	Mid (B)	+ (+)
PHM2_046		Mid (B)	Mid (C)	+ (–)
PHM2_047		Mid (C)	Mid (C)	+ (+)
PHM2_048		Mid (B)	Mid (B)	+ (+)
PHM2_049		Mid (B)	Mid (B)	+ (+)
PHM2_050		Mid (B)	Mid (B)	+ (+)
PHM2_051		Mid (B)	Mid (B)	+ (+)
PHM2_052		Mid (B)	Mid (B)	+ (+)
PHM2_053		Mid (B)	Mid (B)	+ (+)
PHM2_054		Mid (B)	Mid (B)	+ (+)
PHM2_055		Mid (B)	Mid (B)	+ (+)
PHM2_056		Early (A)	Early (A)	+ (+)
PHM2_057		Early (A)	Early (A)	+ (+)
PHM2_058		Early (A)	Early (A)	+ (+)
PHM2_059		Early (A)	Early (A)	+ (+)
PHM2_060		Early (A)	Early (A)	+ (+)
PHM2_061		Early (A)	Early (A)	+ (+)
PHM2_062		Early (A)	Early (A)	+ (+)
PHM2_063		Early (A)	Early (A)	+ (+)
PHM2_064		Early (A)	Early (A)	+ (+)
PHM2_065		Early (A)	Early (A)	+ (+)
PHM2_066		Early (A)	Early (A)	+ (+)
PHM2_067		Early (A)	Early (A)	+ (+)
PHM2_068		Early (A)	Early (A)	+ (+)
PHM2_069		Early (A)	Early (A)	+ (+)
PHM2_070		Early (A)	Early (A)	+ (+)
PHM2_071		Mid (B)	Early (A)	– (–)
PHM2_072		Early (A)	Early (A)	+ (+)
PHM2_073		Early (A)	Early (A)	+ (+)
PHM2_074		Early (A)	Early (A)	+ (+)
PHM2_075		Mid (B)	Mid (B)	+ (+)
PHM2_076		Mid (B)	Mid (B)	+ (+)
PHM2_077		Mid (B)	Mid (C)	+ (–)
PHM2_078		Mid (B)	Mid (C)	+ (–)
PHM2_079		Mid (B)	Mid (C)	+ (–)
PHM2_080		Mid (C)	Mid (C)	+ (+)
PHM2_081		Late (D)	Late (D)	+ (+)
PHM2_082		Late (D)	Late (D)	+ (+)
PHM2_083		Late (D)	Late (D)	+ (+)
PHM2_084	*g25*	Late (E)	Late (D)	+ (–)
PHM2_085	*g6*	Late (E)	Late (E)	+ (+)
PHM2_086	*g7*	Late (E)	Late (E)	+ (+)
PHM2_087	*g8*	Late (E)	Late (E)	+ (+)
PHM2_088		Late (E)	Late (E)	+ (+)
PHM2_089		Late (E)	Late (E)	+ (+)
PHM2_090		Late (E)	Late (E)	+ (+)
PHM2_091		Late (E)	Late (E)	+ (+)
PHM2_092		Late (E)	Late (E)	+ (+)
PHM2_093		Late (E)	Late (E)	+ (+)
PHM2_094	*g7*	Late (E)	Late (E)	+ (+)
PHM2_095		Late (E)	Late (E)	+ (+)
PHM2_096		Late (E)	Late (E)	+ (+)
PHM2_097		Late (E)	Late (E)	+ (+)
PHM2_098		Late (E)	Late (E)	+ (+)
PHM2_099		Late (D)	Late (D)	+ (+)
PHM2_100		Late (D)	Late (D)	+ (+)
PHM2_101		Late (D)	Late (D)	+ (+)
PHM2_102		Late (E)	Late (D)	+ (–)
PHM2_103		Late (E)	Late (E)	+ (+)
PHM2_104		Mid (C)	Mid (C)	+ (+)
PHM2_105		Mid (C)	Mid (C)	+ (+)
PHM2_106		Late (E)	Late (E)	+ (+)
PHM2_107	*g13*	Late (E)	Late (E)	+ (+)
PHM2_108	*g14*	Late (E)	Late (E)	+ (+)
PHM2_109	*g15*	Late (E)	Late (E)	+ (+)
PHM2_110	*g16*	Late (E)	Late (E)	+ (+)
PHM2_111		Late (E)	Late (E)	+ (+)
PHM2_112		Late (D)	Late (D)	+ (+)
PHM2_113		Late (D)	Mid (C)	– (–)
PHM2_114		Late (D)	Mid (C)	– (–)
PHM2_115	*g17*	Late (D)	Mid (C)	– (–)
PHM2_116	*g18*	Late (E)	Late (E)	+ (+)
PHM2_117	*g19*	Late (E)	Late (E)	+ (+)
PHM2_118	*g20*	Late (D)	Late (D)	+ (+)
PHM2_119		Late (E)	Late (D)	+ (–)
PHM2_120	*g21*	Late (E)	Late (D)	+ (–)
PHM2_121	*g22*	Late (E)	Late (E)	+ (+)
PHM2_122	*g23*	Late (E)	Late (E)	+ (+)
PHM2_123		Mid (C)	Late (D)	– (–)
PHM2_124		Late (D)	Late (D)	+ (+)
PHM2_125	*g3*	Late (D)	Late (D)	+ (+)
PHM2_126	*uvsY*	Late (D)	Late (D)	+ (+)
PHM2_127	*uvsW*	Late (D)	Late (D)	+ (+)
PHM2_128		Late (D)	Late (D)	+ (+)
PHM2_129	*g55*	Mid (C)	Mid (C)	+ (+)
PHM2_130		Mid (C)	Mid (C)	+ (+)
PHM2_131	*g47*	Mid (C)	Mid (C)	+ (+)
PHM2_132		Mid (C)	Late (D)	– (–)
PHM2_133	*g46*	Mid (C)	Late (D)	– (–)
PHM2_134		Mid (B)	Mid (B)	+ (+)
PHM2_135		Early (A)	Mid (B)	– (–)
PHM2_136	*cobS*	Early (A)	Mid (B)	– (–)
PHM2_137		Mid (B)	Mid (B)	+ (+)
PHM2_138	*g45*	Mid (B)	Mid (C)	+ (–)
PHM2_139		Mid (B)	Mid (C)	+ (–)
PHM2_140	*g44*	Mid (C)	Mid (C)	+ (+)
PHM2_141		Mid (B)	Mid (C)	+ (–)
PHM2_142		Mid (B)	Mid (C)	+ (–)
PHM2_143	*dam*	Mid (C)	Mid (C)	+ (+)
PHM2_144		Mid (C)	Mid (C)	+ (+)
PHM2_145	*g62*	Mid (C)	Mid (C)	+ (+)
PHM2_146	*regA*	Late (D)	Late (D)	+ (+)
PHM2_147	*hsp20*	Mid (C)	Mid (C)	+ (+)
PHM2_148		Mid (B)	Mid (B)	+ (+)
PHM2_149	*dam*	Mid (C)	Mid (C)	+ (+)
PHM2_150		Mid (C)	Mid (C)	+ (+)
PHM2_151		Mid (C)	Mid (C)	+ (+)
PHM2_152		Mid (C)	Mid (C)	+ (+)
PHM2_153		Mid (B)	Mid (C)	+ (–)
PHM2_154	*g43*	Mid (C)	Mid (C)	+ (+)
PHM2_155	*uvsX*	Mid (C)	Mid (C)	+ (+)
PHM2_156		Mid (C)	Mid (C)	+ (+)
PHM2_157	*g41*	Mid (C)	Mid (C)	+ (+)
PHM2_158	*hn*	Mid (C)	Mid (C)	+ (+)
PHM2_159	*mazG*	Mid (C)	Mid (C)	+ (+)
PHM2_160		Mid (C)	Mid (C)	+ (+)
PHM2_161		Late (D)	Mid (C)	– (–)
PHM2_162		Late (D)	Late (D)	+ (+)
PHM2_163		Late (D)	Late (E)	+ (–)
PHM2_164		Late (E)	Late (E)	+ (+)
PHM2_165		Late (D)	Late (D)	+ (+)
PHM2_166		Mid (C)	Mid (C)	+ (+)
PHM2_167	*tlmH*	Mid (C)	Mid (C)	+ (+)
PHM2_168		Late (D)	Late (D)	+ (+)
PHM2_169		Late (E)	Late (D)	+ (–)
PHM2_170		Late (E)	Late (D)	+ (–)
PHM2_171		Late (D)	Late (D)	+ (+)
PHM2_172		Mid (C)	Mid (C)	+ (+)
PHM2_173		Mid (B)	Mid (C)	+ (–)
PHM2_174	*g61*	Mid (C)	Mid (C)	+ (+)
PHM2_175	*nrdA*	Mid (C)	Mid (C)	+ (+)
PHM2_176	*nrdB*	Mid (C)	Mid (C)	+ (+)
PHM2_177		Mid (C)	Mid (C)	+ (+)
PHM2_178		Mid (C)	Mid (C)	+ (+)
PHM2_179		Mid (C)	Late (D)	– (–)
PHM2_180		Mid (C)	Late (D)	– (–)
PHM2_181		Mid (B)	Late (D)	– (–)
PHM2_182		Mid (C)	Mid (C)	+ (+)
PHM2_183		Mid (C)	Mid (C)	+ (+)
PHM2_184		Mid (C)	Mid (C)	+ (+)
PHM2_185		Late (D)	Late (D)	+ (+)
PHM2_186		Late (D)	Late (D)	+ (+)
PHM2_187		Mid (C)	Mid (C)	+ (+)
PHM2_188		Mid (B)	Mid (B)	+ (+)
PHM2_189		Mid (B)	Mid (B)	+ (+)
PHM2_190		Mid (B)	Mid (C)	+ (–)
PHM2_191		Late (E)	Late (D)	+ (–)
PHM2_192		Late (E)	Late (D)	+ (–)
PHM2_193		Late (D)	Late (D)	+ (+)
PHM2_194		Late (D)	Late (D)	+ (+)
PHM2_195		Mid (C)	Mid (C)	+ (+)
PHM2_196		Late (D)	Mid (C)	– (–)
PHM2_197		Late (E)	Late (E)	+ (+)
PHM2_198	*hli03*	Mid (B)	Mid (B)	+ (+)
PHM2_199	*hli03*	Mid (B)	Mid (C)	+ (–)
PHM2_200	*hli03*	Mid (B)	Mid (B)	+ (+)
PHM2_201	*hli04*	Mid (B)	Mid (B)	+ (+)
PHM2_202		Mid (B)	Mid (C)	+ (–)
PHM2_203		Mid (C)	Mid (C)	+ (+)
PHM2_204		Mid (C)	Mid (C)	+ (+)
PHM2_205		Mid (C)	Mid (C)	+ (+)
PHM2_206		Mid (C)	Mid (C)	+ (+)
PHM2_207		Mid (C)	Mid (C)	+ (+)
PHM2_208		Mid (B)	Mid (C)	+ (–)
PHM2_209		Mid (C)	Mid (C)	+ (+)
PHM2_210		Mid (C)	Mid (C)	+ (+)
PHM2_211		Mid (C)	Mid (C)	+ (+)
PHM2_212	*psbA*	Mid (C)	Mid (C)	+ (+)
PHM2_213	*psbD*	Mid (C)	Mid (C)	+ (+)
PHM2_214		Mid (B)	Mid (B)	+ (+)
PHM2_215	*g7*	Mid (C)	Mid (C)	+ (+)
PHM2_216		Mid (C)	Mid (C)	+ (+)
PHM2_217		Mid (C)	Mid (C)	+ (+)
PHM2_218		Mid (C)	Mid (C)	+ (+)
PHM2_219		Late (D)	Late (D)	+ (+)
PHM2_220		Late (D)	Late (D)	+ (+)
PHM2_221		Mid (C)	Mid (C)	+ (+)
PHM2_222		Mid (C)	Mid (C)	+ (+)
PHM2_223		Mid (C)	Mid (C)	+ (+)
PHM2_224		Mid (C)	Late (D)	– (–)
PHM2_225		Mid (C)	Mid (C)	+ (+)
PHM2_226		Mid (C)	Mid (C)	+ (+)
PHM2_227	*cp12*	Mid (C)	Mid (C)	+ (+)
PHM2_228	*talC*	Mid (C)	Mid (C)	+ (+)
PHM2_229		Mid (B)	Mid (C)	+ (–)
PHM2_230		Mid (C)	Mid (C)	+ (+)
PHM2_231		Mid (C)	Mid (C)	+ (+)
PHM2_232	*nrdC*	Mid (B)	Mid (C)	+ (–)
PHM2_233		Mid (B)	Mid (C)	+ (–)
PHM2_234		Mid (C)	Mid (C)	+ (+)
PHM2_235		Mid (C)	Mid (C)	+ (+)
PHM2_236		Mid (C)	Mid (C)	+ (+)
PHM2_237		Mid (C)	Mid (C)	+ (+)
PHM2_238	*td*	Mid (C)	Mid (C)	+ (+)
PHM2_239		Mid (C)	Mid (C)	+ (+)
PHM2_240	*phoH*	Mid (C)	Mid (C)	+ (+)
PHM2_241		Mid (B)	Mid (C)	+ (–)
PHM2_242	*g33*	Mid (B)	Mid (C)	+ (–)
