# Supplementary material for: Gene Expression Patterns during Light and Dark Infection of Prochlorococcus by Cyanophage
Source: PLoS One. 2016 Oct 27;11(10):e0165375. doi: 10.1371/journal.pone.0165375 (PMC5082946; doi:10.1371/journal.pone.0165375)
Supplement: S3 Table — RPKM-normalized counts and log2(fold change) are given for infected relative to uninfected duplicates (NOISeq) in light (Part A) and dark (Part B). NCBI locus tags for Prochlorococcus MED4 are provided. DEGs listed are those detected by both NOISeq and DESeq2 (S1 Table), with an absolute value of log2(fold change) ≥0.4 and total counts at that time point ≥500. Hypothetical proteins are excluded. (PDF) [file pone.0165375.s007.pdf]

S3 Table (Part A)

Locus tag	Counts (RPKM)		Fold ch.	Gene name and function	Pathway
	Inf.	Uninf.	($\log_2$)		
				**** Light: 0.5 h post-inoculation (phage or spent medium) ****	
PMM1385	4269	1356	1.654 ↑	*hli11*, High light inducible protein	Light stress response
PMM1404	837	292	1.521 ↑	*hli5*, High light inducible protein	Light stress response
PMM1135	1401	500	1.487 ↑	*hli14*, High light inducible protein	Light stress response
PMM0336	1465	587	1.320 ↑	Plastoquinol terminal oxidase (PTOX)	Photosynthetic electron transport
PMM1399	774	347	1.156 ↑	*hli6*, High light inducible protein	Light stress response
PMM1398	744	379	0.972 ↑	*hli7*, High light inducible protein	Light stress response
PMM1629	1791	1124	0.672 ↑	Cyanobacteria-specific RpoD-like sigma factor, type-6	Transcription
PMM1663	588	910	–0.631 ↓	Photosystem I assembly related protein Ycf37	Photosynthesis: PSI
				**** Light: 1.5 h post-inoculation (phage or spent medium) ****	
PMM0814	1820	1086	0.744 ↑	Cytochrome *c* oxidase subunit VIb-like	Photosynthetic electron transport
RNA_15	272	551	–1.021 ↓	tRNA-Arg2, tRNA-Arg-CCG	Protein translation
PMM0510	1266	2068	–0.708 ↓	Possible reverse transcriptase, RNA-dependent	Reverse transcription
PMM0573	4844	7419	–0.615 ↓	AbrB family transcriptional regulator	Transcriptional regulation
PMM0290	218	320	–0.558 ↓	Possible ABC transporter, ATP-binding component	Transport
PMM1309	533	771	–0.533 ↓	*ftsZ*, Cell division protein FtsZ (EC 3.4.24.-)	Cell division
				**** Light: 2.5 h post-inoculation (phage or spent medium) ****	
PMM0223	25985	18992	0.452 ↑	*psbA*, Photosystem II protein D1 (PsbA)	Photosynthesis: PSII
PMM1167	297	510	–0.778 ↓	Macrophage migration inhibitory factor family	(unknown)
PMM1568	1580	2424	–0.617 ↓	Hypothetical membrane protein	Membrane proteins
				**** Light: 4.5 h post-inoculation (phage or spent medium) ****	
PMM0272	6299	3626	0.797 ↑	*psbK*, Photosystem II protein PsbK	Photosynthesis: PSII
PMM0573	3204	4931	–0.622 ↓	AbrB family transcriptional regulator (part 2)	Transcriptional regulation
PMM1148	2821	3981	–0.497 ↓	Possible 7kD DNA-binding domain	DNA-binding domains
				**** Light: 8.5 h post-inoculation (phage or spent medium) ****	
PMM0272	6608	3754	0.816 ↑	*psbK*, Photosystem II protein PsbK	Photosynthesis: PSII
RNA_17	1806	1051	0.781 ↑	tRNA-Val2, tRNA-Val-TAC	Protein translation
PMM0223	27918	19335	0.530 ↑	*psbA*, Photosystem II protein D1 (PsbA)	Photosynthesis: PSII
PMM1402	203	452	–1.152 ↓	Ferredoxin-NADP(+) reductase (EC 1.18.1.2) (FNR)	Photosynthetic electron transport
PMM0987	1481	3102	–1.067 ↓	*rpsU*, SSU ribosomal protein S21p	Ribosome
PMM1007	218	415	–0.928 ↓	Cell division protein FtsK	Cell division
PMM1183	2051	3476	–0.761 ↓	*rpmH*, LSU ribosomal protein L34p	Ribosome
PMM0870	3918	6476	–0.725 ↓	*rpmG*, LSU ribosomal protein L33p	Ribosome
PMM0732	657	1048	–0.674 ↓	Possible major surface glycoprotein	Cell surface glycoproteins
RNA_8	1792	2774	–0.630 ↓	tRNA-Lys1, tRNA-Lys-TTT	Protein translation
PMM0870	4417	6814	–0.625 ↓	*rpmG*, SSU ribosomal protein S18p	Ribosome
PMM0500	602	920	–0.611 ↓	Cyanobacterial protein slr0575	(unknown)
PMM1171	766	1160	–0.598 ↓	*isiB*, Flavodoxin 1	Photosynthetic electron transport
PMM1655	428	648	–0.598 ↓	*tig*, Cell division trigger factor (EC 5.2.1.8)	Cell division
PMM1344	1691	2522	–0.577 ↓	*rplU*, LSU ribosomal protein L21p	Ribosome
PMM0943	2555	3798	–0.572 ↓	*rpsO*, SSU ribosomal protein S15p (S13e)	Ribosome

S3 Table (Part B)

Locus tag	Counts (RPKM)		Fold ch.	Gene name and function	Pathway
	Inf.	Uninf.	($\log_2$)		
				**** Dark: 0.5 h post-inoculation (phage or spent medium) ****	
No differentially expressed genes detected above thresholds					
				**** Dark: 1.5 h post-inoculation (phage or spent medium) ****	
No differentially expressed genes detected above thresholds					
				**** Dark: 2.5 h post-inoculation (phage or spent medium) ****	
PMM0263	33247	21973	0.597 ↑	*amt1*, Ammonium transporter family	Transport
RNA_15	178	483	–1.435 ↓	tRNA-Arg2, tRNA-Arg-CCG	Protein translation
PMM0689	283	634	–1.164 ↓	*hli22*, High light inducbile protein	Light stress response
RNA_20	198	426	–1.106 ↓	tRNA-Ser1, tRNA-Ser-GCT	Protein translation
PMM0740	401	839	–1.065 ↓	*petN*, Cytochrome $b_6 f$ complex subunit VIII	Photosynthetic electron transport
PMM0686	295	601	–1.029 ↓	*clpS*, ATP-dependent Clp protease adaptor protein ClpS	Proteolysis
PMM0814	832	1686	–1.019 ↓	Cytochrome *c* oxidase subunit VIb-like	Photosynthetic electron transport
PMM1503	250	495	–0.986 ↓	Possible NDP-sugar pyrophosphorylase, LPS biosynthesis	Lipopolysaccharide biosynthesis
PMM1588	269	527	–0.971 ↓	Possible conserved carboxylase domain	(unknown)
				**** Dark: 4.5 h post-inoculation (phage or spent medium) ****	
PMM0806	1559	933	0.740 ↑	Bacterial regulatory proteins, Crp family	Transcriptional regulation
PMM0941	3461	2090	0.727 ↑	Possible cAMP phosphodiesterases class-II precursor	Signal transduction
RNA_3	19142	12513	0.613 ↑	tRNA-Ile1, tRNA-Ile-GAT	Protein translation
RNA_4	10701	7789	0.458 ↑	tRNA-Ala1, tRNA-Ala-TGC	Protein translation
PMM0263	29002	21119	0.458 ↑	*amt1*, Ammonium transporter family	Transport
PMM0689	151	371	–1.294 ↓	*hli22*, High light inducbile protein	Light stress response
PMM1400	1763	3777	–1.099 ↓	Possible hemagglutinin-neuraminidase	Viral proteins
PMM1122	599	1237	–1.046 ↓	*apt*, Adenine phosphoribosyltransferase (EC 2.4.2.7)	Purine metabolism
PMM0814	1071	1970	–0.879 ↓	Cytochrome *c* oxidase subunit VIb-like	Photosynthetic electron transport
RNA_16	214	389	–0.864 ↓	tRNA-Cys1, tRNA-Cys-GCA	Protein translation
PMM1663	924	1645	–0.832 ↓	Photosystem I assembly related protein Ycf37	Photosynthesis: PSI
PMM0691	209	370	–0.824 ↓	Tryptophan-rich protein DUF2389, Ssr2843 homolog	(unknown)
PMM0747	205	360	–0.812 ↓	*pcyA*, Phycocyanobilin:ferredoxin oxidoreductase (EC 1.3.7.5)	Porphyrin and chlorophyll metabolism
PMM1437	4805	8345	–0.796 ↓	*groES*, Heat shock protein 60 family co-chaperone GroES	Molecular chaperones
PMM0949	195	338	–0.795 ↓	COG1939: Ribonuclease III family protein	Nucleases
PMM2011	271	466	–0.781 ↓	Possible chorismate binding enzyme	(unknown)
PMM1181	209	358	–0.775 ↓	*tyrA*, Chorismate mutase II (EC 5.4.99.5)	Phe, Tyr and Trp biosynthesis
PMM1459	258	441	–0.771 ↓	*ccdA*, Cytochrome *c*-type biogenesis protein CcdA	Photosynthetic electron transport
PMM0686	460	777	–0.756 ↓	ATP-dependent Clp protease adaptor protein ClpS	Proteolysis
				**** Dark: 8.5 h post-inoculation (phage or spent medium) ****	
PMM0806	1652	1046	0.659 ↑	Bacterial regulatory proteins, Crp family	Transcriptional regulation
RNA_4	12439	8693	0.517 ↑	tRNA-Ala1, tRNA-Ala-TGC	Protein translation
PMM1119	4183	2991	0.484 ↑	*som*, Possible porin	Transport
PMM1121	8685	6262	0.472 ↑	*som*, Possible porin	Transport
PMM0263	17076	12368	0.465 ↑	*amt1*, Ammonium transporter family	Transport
RNA_41	4234	3140	0.431 ↑	5S RNA	Protein translation
PMM1402	393	681	–0.794 ↓	Ferredoxin-NADP(+) reductase (EC 1.18.1.2) (FNR)	Photosynthetic electron transport
PMM1629	739	1246	–0.753 ↓	Cyanobacteria-specific RpoD-like sigma factor, type-6	Transcription
