# Supplementary material for: Gene Expression Patterns during Light and Dark Infection of Prochlorococcus by Cyanophage
Source: PLoS One. 2016 Oct 27;11(10):e0165375. doi: 10.1371/journal.pone.0165375 (PMC5082946; doi:10.1371/journal.pone.0165375)
Supplement: S4 Table — RPKM-normalized counts and log2(fold change) are given for dark relative to light duplicates (NOISeq) in uninfected (Part A) and infected (Part B). NCBI locus tags for Prochlorococcus MED4 are provided. DEGs listed are those detected by both NOISeq and DESeq2 (S1 Table), with an absolute value of log2(fold change) ≥1.5 and total counts at that time point ≥500. Hypothetical proteins are excluded. (PDF) [file pone.0165375.s008.pdf]

S4 Table (Part A)

Locus tag	Counts (RPKM)		Fold ch.	Gene name and function	Pathway
	Dark	Light	(log_2)		
				*** *Uninfected: 0.5 h post-inoculation (spent medium)* ***	
No differentially expressed genes detected above thresholds					
				*** *Uninfected: 1.5 h post-inoculation (spent medium)* ***	
PMM0356	10999	2995	1.877 ↑	Proline iminopeptidase (EC 3.4.11.5)	Arginine and proline metabolism
				*** *Uninfected: 2.5 h post-inoculation (spent medium)* ***	
PMM0356	14094	1490	3.242 ↑	Proline iminopeptidase (EC 3.4.11.5)	Arginine and proline metabolism
PMM1176	1696	550	1.626 ↑	Possible Helix-turn-helix protein, CopG family	Transcriptional regulation
PMM1289	214	640	–1.582 ↓	Cyanobacteria-specific RpoD-like sigma factor, type-12	RNA polymerase
				*** *Uninfected: 4.5 h post-inoculation (spent medium)* ***	
PMM0356	11503	1484	2.955 ↑	Proline iminopeptidase (EC 3.4.11.5)	Arginine and proline metabolism
PMM1176	2387	370	2.691 ↑	Possible Helix-turn-helix protein, CopG family	Transcriptional regulation
PMM1058	490	94	2.379 ↑	*petG*, Cytochrome b6-f complex subunit V (PetG)	Photosynthesis
PMM1400	3777	1174	1.686 ↑	Possible hemagglutinin-neuraminidase	Viral proteins
PMM1179	386	123	1.649 ↑	Permease of the drug/metabolite transporter (DMT) superfamily	Transport
PMM1289	86	682	–2.993 ↓	Cyanobacteria-specific RpoD-like sigma factor, type-12	RNA polymerase
PMM1309	164	749	–2.192 ↓	*ftsZ*, Cell division protein FtsZ (EC 3.4.24.-)	Cell division
PMM0502	158	626	–1.984 ↓	Single-stranded DNA-binding protein	Replication
PMM0577	271	974	–1.844 ↓	Cyanobacteria-specific RpoD-like sigma factor, type-14	Transcriptional regulation
PMM0244	315	1074	–1.768 ↓	*dcd*, Deoxycytidine triphosphate deaminase (EC 3.5.4.13)	Pyrimidine metabolism
PMM1711	292	902	–1.629 ↓	*sps*, Sucrose phosphate synthase(EC:2.4.1.14)	Starch and sucrose metabolism
PMM0245	276	832	–1.594 ↓	cob(I)alamin adenosyltransferase	Porphyrin and chlorophyll metabolism
PMM1697	462	1367	–1.565 ↓	Cyanobacteria-specific RpoD-like sigma factor, type-13	RNA polymerase
PMM0243	226	663	–1.549 ↓	*thyX*, Thymidylate synthase thyX (EC 2.1.1.-)	Pyrimidine metabolism
				*** *Uninfected: 8.5 h post-inoculation (spent medium)* ***	
PMM0356	14936	1565	3.255 ↑	Proline iminopeptidase (EC 3.4.11.5)	Arginine and proline metabolism
PMM1176	2905	391	2.893 ↑	Possible Helix-turn-helix protein, CopG family	Transcriptional regulation
PMM1058	1619	229	2.82 ↑	*petG*, Ribosomal RNA small subunit methyltransferase D (EC 2.1.1.-)	Photosynthesis
PMM1179	498	151	1.722 ↑	Permease of the drug/metabolite transporter (DMT) superfamily	Transport
PMM0747	489	170	1.52 ↑	*pcyA*, Phycocyanobilin:ferredoxin oxidoreductase (EC 1.3.7.5)	Porphyrin and chlorophyll metabolism
PMM1289	72	809	–3.487 ↓	Cyanobacteria-specific RpoD-like sigma factor, type-12	RNA polymerase
PMM1309	62	637	–3.355 ↓	*ftsZ*, Cell division protein FtsZ (EC 3.4.24.-)	Cell division
PMM0577	122	1109	–3.18 ↓	Cyanobacteria-specific RpoD-like sigma factor, type-14	Transcriptional regulation
PMM0245	112	846	–2.917 ↓	cob(I)alamin adenosyltransferase	Porphyrin and chlorophyll metabolism
PMM1711	159	1051	–2.724 ↓	*sps*, Sucrose phosphate synthase(EC:2.4.1.14)	Starch and sucrose metabolism
PMM0244	154	1010	–2.711 ↓	*dcd*, Deoxycytidine triphosphate deaminase (EC 3.5.4.13)	Pyrimidine metabolism
PMM0502	93	530	–2.514 ↓	Single-stranded DNA-binding protein	Replication
PMM0243	124	570	–2.196 ↓	*thyX*, Thymidylate synthase thyX (EC 2.1.1.-)	Pyrimidine metabolism
PMM0330	100	456	–2.19 ↓	Possible LysM domain	Cell membrane
PMM0779	112	482	–2.11 ↓	*purS*, Phosphoribosylformylglycinamidine synthase (EC 6.3.5.3)	Purine metabolism
PMM0661	262	1109	–2.081 ↓	*nrdJ*, Ribonucleotide reductase class II (B12-dependent) (EC 1.17.4.1)	Purine and pyrimidine metabolism
PMM0576	131	549	–2.069 ↓	COG3339 conserved hypothetical protein	(unknown)
PMM0660	802	3322	–2.051 ↓	Possible RNA recognition motif (RRM)	Translational regulation
PMM0519	363	1410	–1.958 ↓	*talB*, Transaldolase (EC 2.2.1.2)	Pentose phosphate pathway
PMM0970	297	1109	–1.899 ↓	*urtA*, Urea ABC transporter, substrate binding protein UrtA	ABC transporters
N/A	147	544	–1.893 ↓	Protein family PM-1	(unknown)
PMM1611	164	599	–1.87 ↓	*thiC*, Thiamin biosynthesis protein ThiC	Thiamine metabolism
PMM1321	771	2759	–1.839 ↓	Bacterial histone-like DNA-binding protein	DNA-binding proteins
PMM1150	223	746	–1.741 ↓	Thioredoxin reductase (EC 1.8.1.9)	Pyrimidine metabolism
PMM0387	165	526	–1.676 ↓	*aroD*, 3-dehydroquinate dehydratase II (EC 4.2.1.10)	Phe, Tyr and Trp biosynthesis
PMM0920	197	624	–1.665 ↓	*glnA*, Glutamine synthetase type I (EC 6.3.1.2)	Alanine, aspartate and glutamate metabolism
PMM1657	391	1181	–1.596 ↓	*clpX*, ATP-dependent Clp protease ATP-binding subunit ClpX	Proteolysis
PMM1148	1281	3829	–1.579 ↓	Possible 7kD DNA-binding domain	DNA-binding proteins
PMM1697	367	1095	–1.579 ↓	Cyanobacteria-specific RpoD-like sigma factor, type-13	RNA polymerase
PMM0258	239	687	–1.52 ↓	*glyA*, Serine hydroxymethyltransferase (EC 2.1.2.1)	Glycine, serine and threonine metabolism
PMM1145	146	413	–1.502 ↓	*pntB*, NAD(P) transhydrogenase subunit beta (EC 1.6.1.2)	Nicotinate and nicotinamide metabolism
PMM1568	888	2513	–1.501 ↓	Hypothetical membrane protein	Membrane proteins

S4 Table (Part B)

Locus tag	Counts (RPKM)		Fold ch.	Gene name and function	Pathway
	Dark	Light	($\log_2$)		
				**** Infected: 0.5 h post-inoculation (phage) ****	
PMM0336	209	1465	–2.806 ↓	Plastoquinol terminal oxidase	Photosynthetic electron transport
PMM0817	110	657	–2.573 ↓	*hli17*, High light inducbile protein	Light stress response
PMM1135	300	1401	–2.226 ↓	*hli14*, High light inducbile protein	Light stress response
PMM1399	177	774	–2.127 ↓	*hli6*, High light inducbile protein	Light stress response
PMM0817	188	768	–2.028 ↓	*hli17*, High light inducbile protein	Light stress response
PMM0815	195	764	–1.972 ↓	*hli19*, High light inducbile protein	Light stress response
PMM1399	154	586	–1.926 ↓	*hli6*, High light inducbile protein	Light stress response
PMM1385	1246	4269	–1.777 ↓	*hli11*, High light inducbile protein	Light stress response
PMM1398	234	744	–1.668 ↓	*hli7*, High light inducbile protein	Light stress response
PMM1404	269	837	–1.639 ↓	*hli5*, High light inducbile protein	Light stress response
				**** Infected: 1.5 h post-inoculation (phage) ****	
PMM0356	10006	1898	2.398 ↑	Proline iminopeptidase (EC 3.4.11.5)	Arginine and proline metabolism
				**** Infected: 2.5 h post-inoculation (phage) ****	
PMM0356	14796	1292	3.518 ↑	Proline iminopeptidase (EC 3.4.11.5)	Arginine and proline metabolism
PMM1289	207	656	–1.666 ↓	Cyanobacteria-specific RpoD-like sigma factor, type-12	RNA polymerase
PMM1539	189	548	–1.533 ↓	*adk*, Adenylate kinase (EC 2.7.4.3)	Purine metabolism
				**** Infected: 4.5 h post-inoculation (phage) ****	
PMM0356	17034	1229	3.793 ↑	Proline iminopeptidase (EC 3.4.11.5)	Arginine and proline metabolism
PMM1176	2236	451	2.311 ↑	Possible Helix-turn-helix protein, CopG family	Transcriptional regulation
PMM0348	2839	888	1.676 ↑	Possible Spectrin repeat	Cytoskeleton
PMM1289	89	707	–2.995 ↓	Cyanobacteria-specific RpoD-like sigma factor, type-12	RNA polymerase
PMM1711	251	1053	–2.07 ↓	*sps*, Sucrose phosphate synthase(EC:2.4.1.14)	Starch and sucrose metabolism
PMM0243	224	863	–1.946 ↓	*thyX*, Thymidylate synthase thyX (EC 2.1.1.-)	Pyrimidine metabolism
PMM0244	352	1286	–1.871 ↓	*dcd*, Deoxycytidine triphosphate deaminase (EC 3.5.4.13)	Pyrimidine metabolism
PMM1309	190	661	–1.801 ↓	*ftsZ*, Cell division protein FtsZ (EC 3.4.24.-)	Cell division
PMM0245	284	924	–1.701 ↓	cob(I)alamin adenosyltransferase	Porphyrin and chlorophyll metabolism
PMM0577	345	1038	–1.587 ↓	Cyanobacteria-specific RpoD-like sigma factor, type-14	Transcriptional regulation

(continued on next page)

S4 Table (Part B) (continued)

Locus tag	Counts (RPKM)		Fold ch.	Gene name and function	Pathway
	Dark	Light	(log_2)		
				**** Infected: 8.5 h post-inoculation (phage) ****	
PMM0356	15827	1379	3.521 ↑	Proline iminopeptidase (EC 3.4.11.5)	Arginine and proline metabolism
PMM1176	2772	517	2.424 ↑	Possible Helix-turn-helix protein, CopG family	Transcriptional regulation
PMM0348	4088	1155	1.824 ↑	Possible Spectrin repeat	Cytoskeleton
PMM1400	3120	1043	1.581 ↑	Possible hemagglutinin-neuraminidase	Viral proteins
PMM1289	72	731	–3.35 ↓	Cyanobacteria-specific RpoD-like sigma factor, type-12	RNA polymerase
PMM1309	67	597	–3.145 ↓	*ftsZ*, Cell division protein FtsZ (EC 3.4.24.-)	Cell division
PMM0577	140	1113	–2.994 ↓	Cyanobacteria-specific RpoD-like sigma factor, type-14	Transcriptional regulation
PMM0245	115	895	–2.957 ↓	cob(I)alamin adenosyltransferase	Porphyrin and chlorophyll metabolism
PMM0244	165	1232	–2.9 ↓	*dcd*, Deoxycytidine triphosphate deaminase (EC 3.5.4.13)	Pyrimidine metabolism
PMM1711	168	1251	–2.896 ↓	*sps*, Sucrose phosphate synthase(EC:2.4.1.14)	Starch and sucrose metabolism
PMM0243	136	725	–2.412 ↓	*thyX*, Thymidylate synthase thyX (EC 2.1.1.-)	Pyrimidine metabolism
PMM0502	99	523	–2.403 ↓	Single-stranded DNA-binding protein	Replication
PMM1321	663	2902	–2.131 ↓	Bacterial histone-like DNA-binding protein	DNA-binding proteins
PMM1145	112	445	–1.99 ↓	*pntB*, NAD(P) transhydrogenase subunit beta (EC 1.6.1.2)	Nicotinate and nicotinamide metabolism
PMM0661	260	1014	–1.96 ↓	*nrdJ*, Ribonucleotide reductase class II (B12-dependent) (EC 1.17.4.1)	Purine and pyrimidine metabolism
RNA_15	107	409	–1.938 ↓	tRNA-Arg2, tRNA-Arg-CCG	Protein translation
PMM0576	124	474	–1.936 ↓	COG3339 conserved hypothetical protein	(unknown)
PMM0779	123	469	–1.929 ↓	*purS*, Phosphoribosylformylglycinamidine synthase (EC 6.3.5.3)	Purine metabolism
PMM1697	308	1134	–1.879 ↓	Cyanobacteria-specific RpoD-like sigma factor, type-13	RNA polymerase
PMM1146	212	746	–1.815 ↓	PntA-2, NAD(P) transhydrogenase alpha subunit (EC 1.6.1.2)	Nicotinate and nicotinamide metabolism
PMM1380	184	624	–1.761 ↓	HAD-superfamily hydrolase, subfamily IA, variant 3	Hydrolase (unknown)
PMM1150	211	694	–1.719 ↓	Thioredoxin reductase (EC 1.8.1.9)	Pyrimidine metabolism
PMM0258	214	690	–1.692 ↓	*glyA*, Serine hydroxymethyltransferase (EC 2.1.2.1)	Glycine, serine and threonine metabolism
PMM0660	810	2438	–1.59 ↓	Possible RNA recognition motif (RRM)	Translational regulation
N/A	267	804	–1.589 ↓	tRNA-Leu3, tRNA-Leu-CAA	Protein translation
PMM1611	189	549	–1.536 ↓	*thiC*, Thiamin biosynthesis protein ThiC	Thiamine metabolism
PMM0740	362	1041	–1.524 ↓	*petN*, Cytochrome b6f complex subunit VIII	Photosynthesis
PMM0117	404	1158	–1.52 ↓	TPR-repeat protein, specific for cyanobacteria	(unknown)
PMM0128	356	1018	–1.515 ↓	*rpaA*, Two-component system response regulator	Two-component system
PMM0970	353	1010	–1.515 ↓	*urtA*, Urea ABC transporter, substrate binding protein UrtA	ABC transporters
PMM0444	186	531	–1.514 ↓	*ctaE*, Cytochrome c oxidase polypeptide III (EC 1.9.3.1)	Oxidative phosphorylation
