# Supplementary material for: Gene Expression Patterns during Light and Dark Infection of Prochlorococcus by Cyanophage
Source: PLoS One. 2016 Oct 27;11(10):e0165375. doi: 10.1371/journal.pone.0165375 (PMC5082946; doi:10.1371/journal.pone.0165375)
Supplement: S5 Table — RPKM-normalized counts and log2(fold change) are given for infected relative to uninfected duplicates (NOISeq) in light (Part A) and dark (Part B). NCBI locus tags for Prochlorococcus MED4 are provided. DEGs listed are those detected by both NOISeq and DESeq2 (S1 Table), with an absolute value of log2(fold change) ≥0.4 and total counts at that time point ≥100. Hypothetical proteins are excluded in Part B. (PDF) [file pone.0165375.s009.pdf]

S5 Table (Part A)

Locus tag	Counts (RPKM)		Fold ch.	Gene name and function	Pathway
	Inf.	Uninf.	($\log_2$)		
			*** *Antisense: Light: 0.5 h post-inoculation (phage or spent medium)* ***		
PMM1628	2638	1537	0.780 ↑	Possible alpha/beta hydrolase superfamily	(unknown)
PMM1318	2859	4892	–0.775 ↓	FIG00940231: hypothetical protein	(unknown)
PMM1112	1569	2471	–0.655 ↓	FIG00940583: hypothetical protein	(unknown)
N/A	9428	14496	–0.621 ↓	FIG00942324: hypothetical protein	(unknown)
PMM0659	12615	18232	–0.531 ↓	DNA ligase (EC 6.5.1.2)	DNA replication and repair
PMM1439	5579	7850	–0.493 ↓	*atpC*, ATP synthase epsilon chain (EC 3.6.3.14)	Oxidative phosphorylation
			*** *Antisense: Light: 1.5 h post-inoculation (phage or spent medium)* ***		
PMM1130	471	150	1.648 ↑	Hypothetical protein	(unknown)
PMM0865	994	504	0.979 ↑	FIG00940579: hypothetical protein	(unknown)
N/A	2632	1536	0.777 ↑	FIG00940756: hypothetical protein	(unknown)
			*** *Antisense: Light: 2.5 h post-inoculation (phage or spent medium)* ***		
PMM0223	601	46	3.703 ↑	*psbA*, Photosystem II protein D1 (PsbA)	Photosynthesis
PMM0906	3525	2171	0.699 ↑	*psaK*, photosystem I subunit X (PsaK, PsaK1)	Photosynthesis
			*** *Antisense: Light: 4.5 h post-inoculation (phage or spent medium)* ***		
PMM0223	1194	46	4.711 ↑	*psbA*, Photosystem II protein D1 (PsbA)	Photosynthesis
			*** *Antisense: Light: 8.5 h post-inoculation (phage or spent medium)* ***		
PMM0223	1162	72	4.022 ↑	*psbA*, Photosystem II protein D1 (PsbA)	Photosynthesis
RNA_4	1185	111	3.413 ↑	tRNA-Ala1, tRNA-Ala-TGC	Protein translation
PMM0154	2485	1302	0.932 ↑	Bacterial regulatory protein, LuxR family	Transcriptional regulation
N/A	89646	251387	–1.488 ↓	Hypothetical protein	(unknown)

S5 Table (Part B)

Locus tag	Counts (RPKM)		Fold ch.	Gene name and function	Pathway
	Inf.	Uninf.	(log_2)		
*** *Antisense: Dark: 0.5 h post-inoculation (phage or spent medium)* ***					
No differentially expressed genes detected above thresholds					
*** *Antisense: Dark: 1.5 h post-inoculation (phage or spent medium)* ***					
No differentially expressed genes detected above thresholds					
*** *Antisense: Dark: 2.5 h post-inoculation (phage or spent medium)* ***					
RNA_4	252	35	2.865 ↑	tRNA-Ala1, tRNA-Ala-TGC	Protein translation
PMM0374	2286	1200	0.930 ↑	Twin-arginine translocation protein TatA	Protein export
PMM0143	14947	10861	0.461 ↑	*crtB*/*pys*, Phytoene synthase (EC 2.5.1.32)	Carotenoid biosynthesis
PMM0064	7291	5507	0.405 ↑	*hli2*, High light inducbile protein	Light stress response
PMM1507	16	99	–2.596 ↓	*rpsJ*, SSU ribosomal protein S10p (S20e)	Ribosome
PMM1199	48	150	–1.652 ↓	Putative glycosyl transferase	Glycosyltransferases
PMM0021	32	94	–1.579 ↓	*murB*, UDP-N-acetylenolpyruvoylglucosamine reductase	Peptidoglycan biosynthesis
PMM0892	79	199	–1.341 ↓	*argC*, N-acetyl-gamma-glutamyl-phosphate reductase	Arginine and proline metabolism
PMM0868	41	101	–1.308 ↓	Exoribonuclease II (EC 3.1.13.1)	Nucleases
PMM1625	108	265	–1.293 ↓	*ahcY*, Adenosylhomocysteinase (EC 3.3.1.1)	Cysteine and methionine metabolism
PMM1705	63	154	–1.289 ↓	*aroE*, Shikimate 5-dehydrogenase I alpha (EC 1.1.1.25)	Phe, Tyr and Trp biosynthesis
PMM1390	478	1150	–1.266 ↓	*hli10*, High light inducbile protein	Light stress response
PMM0138	105	244	–1.223 ↓	*plsC*, 1-acyl-sn-glycerol-3-phosphate acyltransferase	Glycerolipid metabolism
PMM1200	96	219	–1.189 ↓	Glycosyl transferase family 2	Glycosyltransferases
PMM1620	158	350	–1.149 ↓	Rod shape-determining protein MreD	Cytoskeleton
PMM0305	343	744	–1.117 ↓	*cpeB*, Phycoerythrin beta chain, Phycobilisome protein	Photosynthesis - antenna proteins
PMM1444	67	143	–1.107 ↓	Small GTP-binding protein domain	Signal transduction
PMM1589	146	293	–1.006 ↓	*purM*/*purG*, Phosphoribosylformylglycinamidine cyclo-ligase	Purine metabolism
PMM1254	167	331	–0.990 ↓	Glycosyl transferase, group 2 family protein	Glycosyltransferases
PMM1053	754	1492	–0.985 ↓	5-formyltetrahydrofolate cyclo-ligase (EC 6.3.3.2)	One-carbon pool by folate
PMM0258	174	340	–0.968 ↓	*glyA*, Serine hydroxymethyltransferase (EC 2.1.2.1)	Glycine, serine and threonine metabolism
PMM0440	157	289	–0.883 ↓	Periplasmic binding protein-like II superfamily	Transport
PMM1126	76	139	–0.871 ↓	Acyltransferase family protein	Cell membrane
PMM0141	220	399	–0.858 ↓	tRNA nucleotidyltransferase, CC-adding (EC 2.7.7.21)	tRNA processing
PMM0043	637	1123	–0.819 ↓	Diflavin flavoprotein SYNW2367	Redox (unknown)
*** *Antisense: Dark: 4.5 h post-inoculation (phage or spent medium)* ***					
PMM0625	85	279	–1.719 ↓	Tryptophan-rich protein DUF2389, Ssr2843 homolog	(unknown)
PMM1692	38	104	–1.440 ↓	Para-aminobenzoate synthase, aminase component	Folate biosynthesis
PMM0948	27	74	–1.433 ↓	*rlmB*, 23S rRNA (guanosine-2'-O-) -methyltransferase	rRNA processing
PMM0868	44	118	–1.414 ↓	Exoribonuclease II (EC 3.1.13.1)	Nucleases
PMM0788	85	213	–1.324 ↓	*proB*, Glutamate 5-kinase (EC 2.7.2.11)	Arginine and proline metabolism
PMM1640	134	322	–1.258 ↓	Acetyltransferase (GNAT) family, Syn7942_2240 homolog	(unknown)
PMM1006	239	553	–1.208 ↓	Glutathione peroxidase (EC 1.11.1.9)	Glutathione metabolism
PMM1138	136	313	–1.202 ↓	GTP-binding and nucleic acid-binding protein YchF	Signal transduction
PMM0882	41	90	–1.134 ↓	*uvrC*, Excinuclease ABC subunit C	Nucleotide excision repair
PMM1647	166	346	–1.062 ↓	DNA polymerase III delta subunit (EC 2.7.7.7)	DNA replication
PMM1628	857	1655	–0.950 ↓	Possible alpha/beta hydrolase superfamily	(unknown)
*** *Antisense: Dark: 8.5 h post-inoculation (phage or spent medium)* ***					
PMM1628	1283	2501	–0.963 ↓	Possible alpha/beta hydrolase superfamily	(unknown)
