# Supplementary material for: Gene Expression Patterns during Light and Dark Infection of Prochlorococcus by Cyanophage
Source: PLoS One. 2016 Oct 27;11(10):e0165375. doi: 10.1371/journal.pone.0165375 (PMC5082946; doi:10.1371/journal.pone.0165375)
Supplement: S6 Table — NCBI locus tags for Prochlorococcus MED4 are provided. Hypothetical proteins are included. (PDF) [file pone.0165375.s010.pdf]

## S6 Table

| Locus tag | Antisense/Sense | Gene name and function |
|---|---|---|
| PMM1518 | 350.97 | FIG00940570: hypothetical protein |
| PMM0661 | 154.70 | Hypothetical protein / 3'-end of class II ribonucleotide reductase NrdJ |
| PMM0337 | 84.46 | FIG00941997: hypothetical protein |
| PMM1378 | 65.59 | *des*/*yocE*, Fatty acid desaturase, type 2 |
| PMED4_15451 | 45.81 | FIG00941715: hypothetical protein |
| PMM0220 | 44.29 | Conserved cyanobacterial protein CP12 |
| PMM0855 | 43.13 | FIG00940706: hypothetical protein |
| PMM0659 | 35.92 | DNA ligase (EC 6.5.1.2) |
| PMM0467 | 33.55 | *gmk*, Guanylate kinase (EC 2.7.4.8) |
| PMED4_12831 | 32.57 | Hypothetical protein |
| RNA_9 | 32.40 | tRNA-Pro2, tRNA-Pro-TGG |
| PMM0014 | 31.95 | tRNA dihydrouridine synthase A |
| RNA_22 | 31.15 | tRNA-Ala2, hypothetical protein |
| PMM0050 | 27.52 | *argJ*, Glutamate N-acetyltransferase (EC 2.3.1.35) / N-acetylglutamate synthase (EC 2.3.1.1) |
| PMM1359 | 25.85 | Predicted membrane protein (COG2259) |
| PMM0350 | 25.82 | Possible TIR domain |
| PMM1058 | 25.19 | *petG*, Ribosomal RNA small subunit methyltransferase D (EC 2.1.1.-) |
| PMM1616 | 23.96 | *smpB*, tmRNA-binding protein SmpB |
| PMM0154 | 23.49 | FIG00941193: hypothetical protein |
| PMM0852 | 20.94 | FIG00940874: hypothetical protein |
| PMM1411 | 17.90 | Hypothetical protein |
| PMM0854 | 16.39 | FIG00944560: hypothetical protein / 5'-end of cell division protein FtsH |
| PMED4_11021 | 16.28 | 3'-end of conserved hypothetical protein |
| PMM0267 | 16.03 | Phospholipase/carboxylesterase family protein, sll1284 homolog |
| PMM1651 | 15.71 | tRNA(Ile)-lysidine synthetase |
| PMM1059 | 14.99 | Ribosomal RNA small subunit methyltransferase D (EC 2.1.1.-) |
| PMM0721 | 14.32 | Possible myosin N-terminal SH3-like domain |
| N/A | 14.15 | FIG00941249: hypothetical protein before PMM0810 |
| PMED4_12821 | 13.22 | FIG00942324: hypothetical protein |
| PMM1322 | 12.89 | *glgX*, Glycogen debranching enzyme (EC 3.2.1.-) |
| N/A | 12.33 | Hypothetical protein between PMM0308 and PMM0309 |
| PMED4_16021 | 11.54 | FIG00944192: hypothetical protein |
| PMM0301 | 11.04 | 5'-methylthioadenosine phosphorylase (EC 2.4.2.28) |
| PMM0928 | 10.46 | Hypothetical protein / 3'-end of preprotein translocase subunit SecF |
