# Supplementary material for: Gene Expression Patterns during Light and Dark Infection of Prochlorococcus by Cyanophage
Source: PLoS One. 2016 Oct 27;11(10):e0165375. doi: 10.1371/journal.pone.0165375 (PMC5082946; doi:10.1371/journal.pone.0165375)
Supplement: S7 Table — Previously conducted experiments have examined the transcriptome of Prochlorococcus MED4 under a diel (light–dark) cycle [43], high-light stress [41], low dissolved ammonium and urea (Tolonen et al., Global gene expression of Prochlorococcus ecotypes in response to changes in nitrogen availability. Mol Syst Biol. 2006;2:53), low dissolved phosphate (Martiny et al., Phosphate acquisition genes in Prochlorococcus ecotypes: Evidence for genome-wide adaptation. Proc Natl Acad Sci USA. 2006;103(33):12552–12557), low dissolved iron [42], low dissolved inorganic carbon [57], and infection by podovirus P-SSP7 in continuous light [21]. Experiments presented here examined the transcriptome of Prochlorococcus MED4 under infection by myovirus P-HM2 in continuous light or following a shift to dark. Plus and minus signs indicate transcripts enriched or diminished in the respective treatment. Brackets around signs indicate transcripts detected as enriched or diminished by only one of NOISeq or DESeq2. (PDF) [file pone.0165375.s011.pdf]

S7 Table

MED4 PMM number	Gene name	Diel cycle [83]		Physicochemical stressors					Phage infection		
		Expr. cluster	Max. mRNA	High [60] light	–N [84]	–P [44]	–Fe [70]	$-CO_2$ [5] ($-O_2$)	P-SSP7 [40] Light	P-HM2 Light	P-HM2 Dark
PMM0093	*hli01*	1	16:00							[–]	[–]
PMM0064	*hli02*	12	4:00								[–]
PMM1482	*hli03*	9	0:00			+		–			[–]
PMM1118	*hli04*	10	1:00	+			+	+			
PMM1404	*hli05*	17	17:00	+			+	+		+	[–]
PMM0818/PMM1399	*hli06*/*hli16*	10	1:00	+			+	+	+	+	[+]
PMM0817/PMM1398	*hli07*/*hli17*	10	1:00	+			+	+	+	+	[+]
PMM0816/PMM1397	*hli08*/*hli18*	10	2:00	+			+	+	+	[+]	
PMM0815/PMM1396	*hli09*/*hli19*	10	2:00	+			+	+	+	+	
PMM1390	*hli10*	4	16:00		+			+			[–]
PMM1385	*hli11*	8	22:00	+				+		+	
PMM1384	*hli12*	8	23:00	+				+		[–]	
PMM1317	*hli13*	3	12:00							[–]	
PMM1135	*hli14*	10	1:00	+			+	+	+	+	[–]
PMM1128	*hli15*	17	8:00	+	+			(+)			[–]
PMM0471	*hli20*	5	16:00								
PMM0690	*hli21*	17	4:00	+	+			+			
PMM0689	*hli22*	11	0:00	+	+			+			–
